# Supplementary material for: Abscission Couples Cell Division to Embryonic Stem Cell Fate
Source: Dev Cell. 2020 Oct 26;55(2):195–208.e5. doi: 10.1016/j.devcel.2020.09.001 (PMC7594744; doi:10.1016/j.devcel.2020.09.001)
Supplement: Document S1. Figures S1–S6 and Tables S1 and S2 [file mmc1.pdf]

Developmental Cell, Volume *55*

Supplemental Information

Abscission Couples Cell Division

to Embryonic Stem Cell Fate

Agathe Chaigne, Céline Labouesse, Ian J. White, Meghan Agnew, Edouard Hannezo, Kevin J. Chalut, and Ewa K. Paluch

Supplementary Information for

Abscission Couples Cell Division to Embryonic Stem Cell Fate

Agathe Chaigne[1,*], Céline Labouesse[2], Ian J. White[1], Meghan Agnew[1], Edouard Hannezo[3], Kevin J. Chalut[2], Ewa K. Paluch[1,2,4,*,#]

[1] MRC Laboratory for Molecular Cell Biology, University College London, London WC1E 6BT, UK.

[2] Wellcome/MRC Cambridge Stem Cell Institute, University of Cambridge, Cambridge CB2 0AW, UK.

[3] Institute of Science and Technology Austria, 3400 Klosterneuburg, Austria.

[4] Department of Physiology, Development and Neuroscience, University of Cambridge, Cambridge CB2 3DY, UK.

* Correspondence: A.C.: a.chaigne@ucl.ac.uk; E.K.P.: ekp25@cam.ac.uk

Lead author

Supplementary Figure Legends

Figure S1. ES cells exit naïve pluripotency after mitosis (related to Figure 1)

A) Representative confocal microscopy images of ES cells expressing REX1-GFP (green) and GAP43-mCherry (magenta), 5h or 40h after being placed in N2B27 supplemented with 6 μM of RO-3306 or equivalent DMSO volume. Scale bars: 10 μm. B) Dot plot showing REX1-GFP intensity 40h after removal of 2i/LIF for cells treated with 6 μM RO-3306 (purple) or DMSO (control, grey). Mean and standard deviation are shown. N=2. C) Dot plot showing proliferation rate over 24h of ES cells treated with DMSO (grey) or 6 μM RO-3306 (purple). Mean and standard deviation are shown. N=5. D) Representative confocal images showing cell areas 40h after 2i/LIF removal and treatment with DMSO (top) or 6 μM RO-3306 (bottom). GAP43-mCherry (magenta) is shown as a membrane marker. One Z-plane is shown around the middle plane of the cells. E) Dot plot showing cell area 40h after removal of 2i/LIF in presence of DMSO (grey) or 6 μM RO-3306 (purple). Mean and standard deviation are shown. N=2. F) Plot of REX1-GFP mean intensity in cells expressing REX1-GFP and GAP43-mCherry during exit from naïve pluripotency, as a function of time. 0h: time of 2i/LIF removal. Mean and standard error of the mean are plotted. N=3, n= 33. G) Bar graphs showing the expression of key pluripotency genes (left) or genes associated with naïve pluripotency exit (right), as assessed by qPCR, during exit from naïve pluripotency. Values are normalized to RNA levels at 0h. The mean and standard error of the mean are shown. N=3. H) Plot of REX1-GFP mean intensity in naïve ES cells expressing REX1-GFP and GAP43-mCherry, as a function of time before and after cell division. The mean and standard error of the mean are plotted. N=2, n= 11 mother cells and 22 daughter cells. I) Left: schematic outlining the analysis of the robustness of the

correlation between time of naïve pluripotency exit and time of division. Right: cumulative probability of correlation coefficients R^2 (see Methods for details) arising at random (purple), plotted together with the experimental correlation (green). All scale bars: 10 μm.

Figure S2. Inducing naïve pluripotency exit early in the cell cycle leads to faster loss of naïve pluripotency (related to Figure 2)

A) Bar graph showing the percentage of cells in each phase of the cell cycle assessed by comparing FUCCI2a fluorescence in cells sorted by cell size; the "small" cell population is compared to the ungated population (G1: red; G1/S: yellow; S/G2/M: green; black: exit of mitosis). Mean and standard error of the mean are plotted. N=2. B) Bar graph showing the percentage of cells in each phase of the cell cycle, as determined by single cell RNA sequencing analysis, 6h after triggering naïve pluripotency exit in cell populations sorted by size (the "early cell cycle" population corresponds to the cells sorted as small, see (A)). G1: red; S: light green; G2/M: green. C) Heat-map showing the Log2 counts of the levels of expression of the main pluripotency genes for the two cell populations obtained by sorting cells by size, and placed in N2B27 for 6h. Early cell cycle (small cells): pink; ungated: blue. D) Bar graph showing the percentage of cells displaying high or low expression levels of *Tfcp2l1* and *Tbx3*, used to assess exit from naïve pluripotency (see Methods). Early cell cycle: pink; ungated: blue.

Figure S3. Confinement induces strongly asymmetric divisions with no effect on exit from naïve pluripotency dynamics (related to Figure 3)

A) Time-lapse spinning-disk confocal microscopy images of a naïve ES cell expressing H2B-RFP (red) and labeled with CellMask^{TM} deep red (cyan) dividing in a 10*10 µm channel (top) or an 8*5 µm channel (bottom). Time in min; 0 min: anaphase. One Z-plane is shown. Scale bar: 10 µm. B) Dot plot representing the sister cell asymmetry ratio (ratio of cell lengths in the channels) of H2B-RFP ES cells dividing in 8*5 µm, 6*8 µm or 10*10 µm channels. Mean and standard deviation are plotted. N=2 for each condition. C) Graph showing an example of REX1 intensity time course for two daughter cells resulting from an asymmetric division in a 8*5 µm microchannel. 0h: time of division.

Figure S4. Additional experiments investigating abscission dynamics during naïve pluripotency exit and the effects of abscission on exiting the ES cell state (Related to Figure 5)

A) Dot plot showing the fraction of cells associated with a midbody remnant (number of midbody remnants divided by number of cells in a given analysis frame) detected in colonies of naïve H2B-RFP ES cells and during exit from naïve pluripotency. Midbody remnants are defined as CRIK spots not attached to a bridge. Mean and standard error of the mean are shown. N=2. B) Dot plot showing the fraction of cells with bridges (number of bridges divided by number of cells in a given analysis frame) as a function of cell density (quantified as the number of cells per frame of fixed dimension) for cells plated on laminin (growing in 2D colonies) at different times of exit from naïve pluripotency. p-value of Pearson correlation <0.0001. C) Box plot showing RNA levels for H2B-RFP ES cells treated with Scrambled siRNA (*SCR*, black) or siRNA against *Alix* (orange) for 24h in 2i/LIF (Naïve, left) or for 24h in 2i/LIF followed by 6h in N2B27 (Exit, right). Values are normalized to *SCR* controls. Mean and

standard error of the mean are shown. N=5 for 2i/LIF and N=2 for 6h after 2i/LIF removal. D) Box plot showing RNA levels for H2B-RFP ES cells treated with Scrambled siRNA (*SCR*, black) or siRNA against *Alix* (orange) for 24h in 2i/LIF followed by 24h in N2B27. Values are normalized to *SCR* controls. Mean and standard error of the mean are shown. N=5. E) Dot plot showing the proliferation (number of cells recovered divided by number of cells plated) after 24h of culture in 2i/LIF for ES cells treated with Scrambled siRNA (*SCR*, black) or siRNA against *Alix* (orange). Mean and standard deviation are plotted. N=7.

Figure S5. Additional experiments showing that interfering with abscission impairs exit form naïve pluripotency (related to Figure 7)

A) Dot plot showing the fraction of cells with bridges (number of bridges divided by number of cells in a given analysis frame) in naïve H2B-RFP ES cell colonies maintained in Serum/LIF or allowed to exit naïve pluripotency for 24 or 48h. Mean and standard error of the mean are shown. N=2. B) Dot plot showing the fraction of cells associated with a midbody remnant (number of midbody remnants divided by number of cells in a given analysis frame) in naïve H2B-RFP ES cell colonies maintained in Serum/LIF or allowed to exit naïve pluripotency for 24 or 48h. Midbody remnants are defined as CRIK spots not attached to a bridge. Mean and standard error of the mean are shown. N=2. C) Box plot showing RNA levels for H2B-RFP ES cells treated with Scrambled siRNA (*SCR*, black) or siRNA against *Alix* (orange) for 24h in Serum/LIF, then cultured in N2B27 for 24h. Values are normalized to *SCR* controls. Mean and standard error of the mean are shown. N=2. D) Schematic showing the relationship between CEP55, ALIX (and ESCRT-I components, such as TSG101), and ESCRT-III. E) Box plot showing RNA levels in naïve cells treated with Scrambled siRNA (*SCR*,

black) or siRNA against *Cep55* (orange) for 24h. *Klf2* levels are shown as a negative control. Values are normalized to *SCR* controls. Mean and standard error of the mean are shown. N=2. F) Dot plot showing the fraction of cells with bridges (number of bridges divided by number of cells in a given analysis frame) for ES cells treated with siRNA *Scrambled* (*SCR*, grey) or *Cep55* (orange), maintained in 2i/LIF or allowed to exit naïve pluripotency for 24 or 48h. Mean and standard error of the mean are shown. N=2. G) Dot plot showing the fraction of cells associated with a midbody remnant (number of midbody remnants divided by number of cells in a given analysis frame) for ES cells treated with siRNA *Scrambled* (*SCR*, grey) or *Cep55* (orange), maintained in 2i/LIF or allowed to exit naïve pluripotency for 24 or 48h. Midbody remnants are defined as CRIK spots not attached to a bridge. Mean and standard error of the mean are shown. N=2. H) Dot plot representing the number of colonies surviving in a clonogenicity assay (see Figure 2A) for ES cells treated with siRNA *Scrambled* (*SCR*, grey) or *Cep55* (orange) for 24h in 2i/LIF, then placed in differentiation-promoting medium for 24h. Mean and standard error of the mean are shown. N=3.

Figure S6. Triggering premature abscission and midbody release accelerates exit from naïve pluripotency (related to Figure 7)

A) Representative example of a bridge laser ablation experiment where the midbody remains connected to one of the daughter cells after ablation. Cells are labeled with SIR-Tubulin. Ablation is highlighted with the red thunderbolt and the location of the midbody with a pink arrowhead. One Z-plane is shown. Scale bar: 10 μm. B) Plot showing REX1-GFP mean intensity for the ablated cells pictured in (A) as a function of time. D2 is the daughter cell retaining the midbody. 0h: time of 2i/LIF removal. Lines

are fitting curves and the time of REX1-GFP downregulation is determined from the first inflexion point (see Methods).

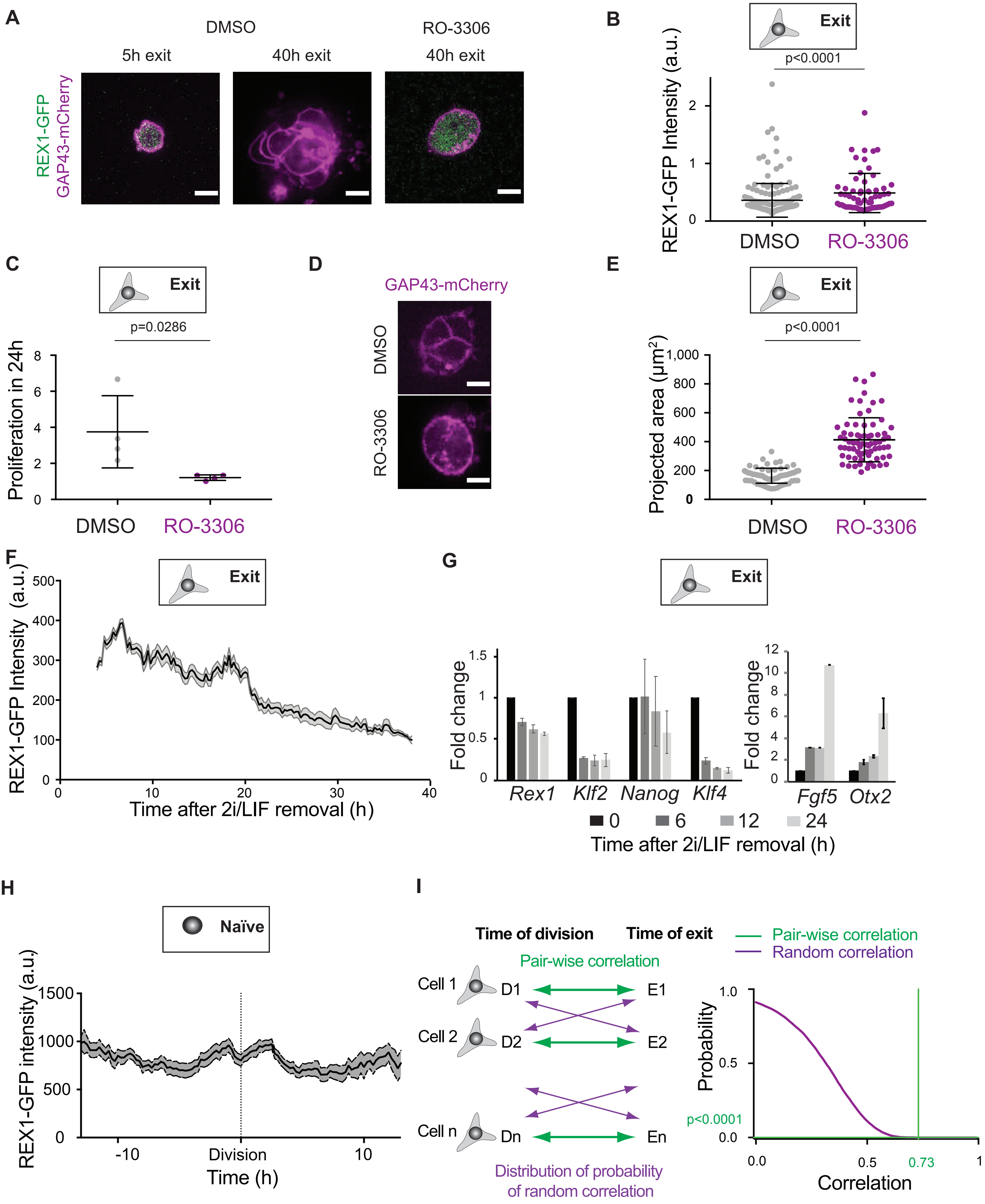
A
DMSO
RO-3306
5h exit
40h exit
40h exit
REX1-GFP
GAP43-mCherry
B
Exit
p<0.0001
REX1-GFP Intensity (a.u.)
DMSO
RO-3306
C
Exit
p=0.0286
Proliferation in 24h
DMSO
RO-3306
D
GAP43-mCherry
DMSO
RO-3306
E
Exit
p<0.0001
Projected area (μm²)
DMSO
RO-3306
F
Exit
REX1-GFP Intensity (a.u.)
Time after 2i/LIF removal (h)
G
Exit
Fold change
Rex1
Klf2
Nanog
Klf4
Fgf5
Otx2
0
6
12
24
Time after 2i/LIF removal (h)
H
Naïve
REX1-GFP intensity (a.u.)
Division
Time (h)
I
Time of division
Time of exit
Pair-wise correlation
Random correlation
Pair-wise correlation
Cell 1
Cell 2
Cell n
D1
D2
Dn
E1
E2
En
Distribution of probability of random correlation
Probability
p<0.0001
0.73
Correlation

Figure S1

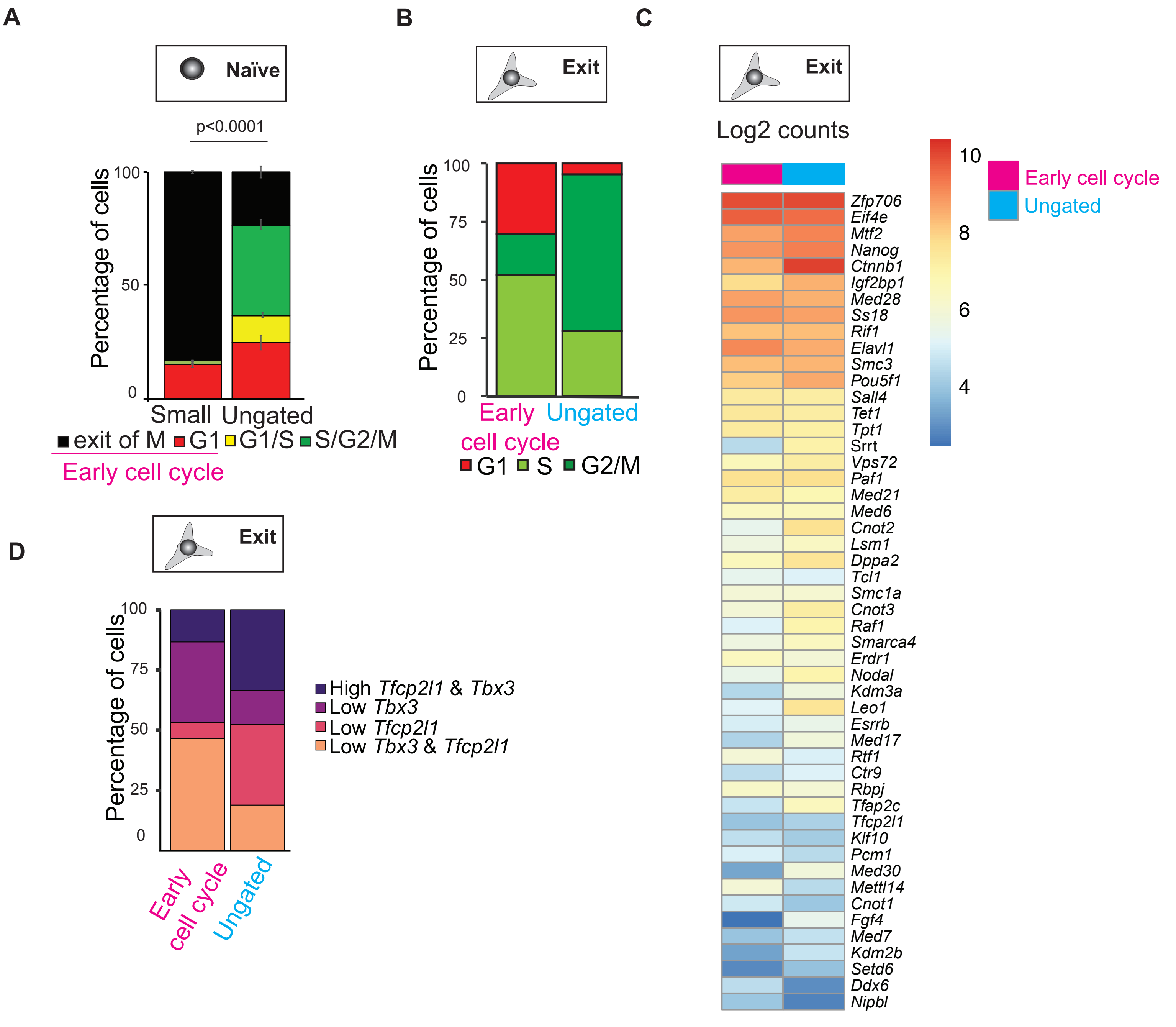
A
Naïve
p<0.0001
Percentage of cells
100
50
0
Small
Ungated
exit of M
G1
G1/S
S/G2/M
Early cell cycle
B
Exit
Percentage of cells
100
75
50
25
0
Early
cell cycle
Ungated
G1
S
G2/M
C
Exit
Log2 counts
10
8
6
4
Early cell cycle
Ungated
Zfp706
Eif4e
Mtf2
Nanog
Ctnnb1
Igf2bp1
Med28
Ss18
Rif1
Elavl1
Smc3
Pou5f1
Sall4
Tet1
Tpt1
Srrt
Vps72
Paf1
Med21
Med6
Cnot2
Lsm1
Dppa2
Tcl1
Smc1a
Cnot3
Raf1
Smarca4
Erdr1
Nodal
Kdm3a
Leo1
Esrrb
Med17
Rtf1
Ctr9
Rbpj
Tfap2c
Tfcp2l1
Klf10
Pcm1
Med30
Mettl14
Cnot1
Fgf4
Med7
Kdm2b
Setd6
Ddx6
Nipbl
D
Exit
Percentage of cells
100
75
50
25
0
High Tfcp2l1 & Tbx3
Low Tbx3
Low Tfcp2l1
Low Tbx3 & Tfcp2l1
Early
cell cycle
Ungated

Figure S2

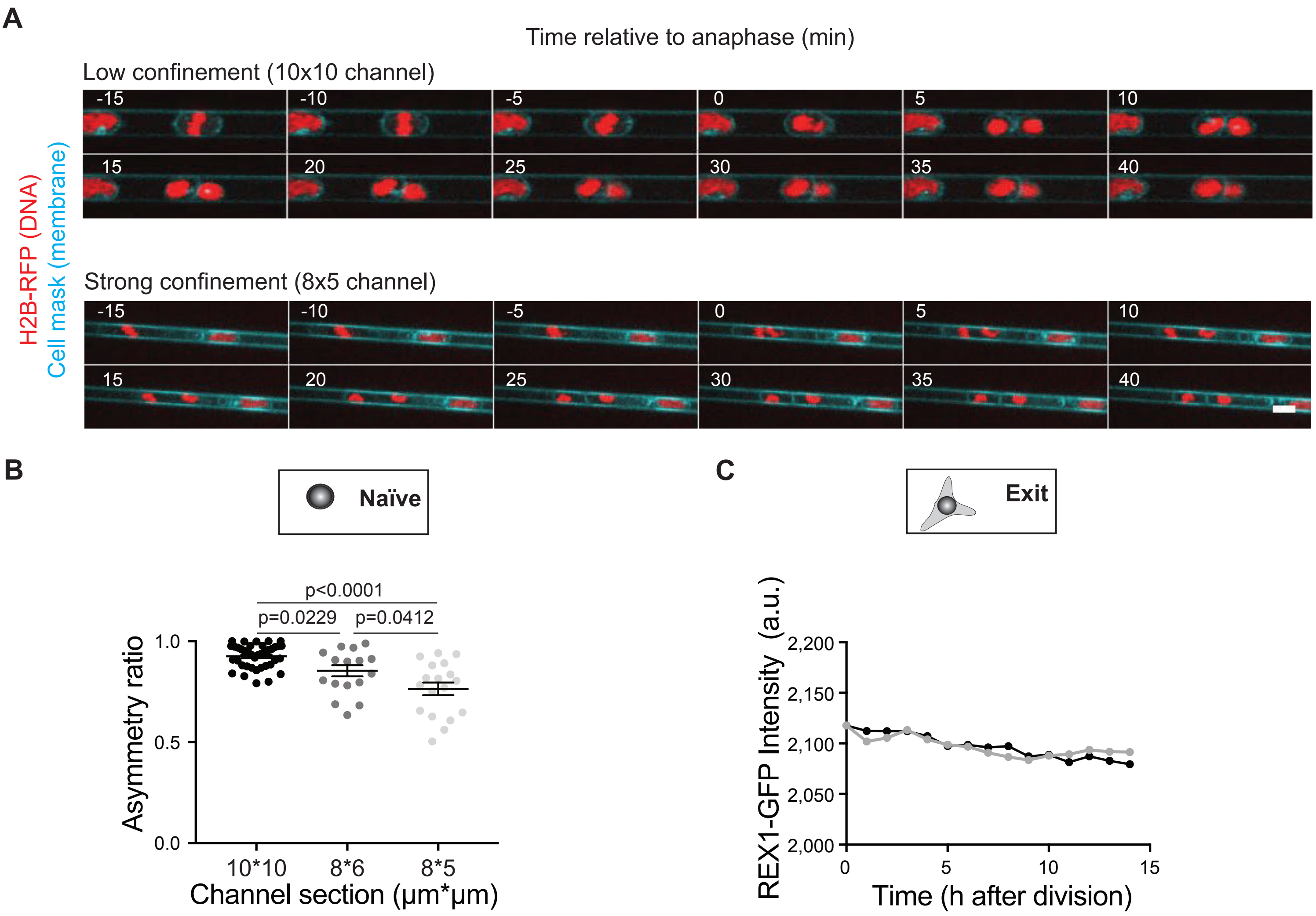
A
Time relative to anaphase (min)
Low confinement (10x10 channel)
-15
-10
-5
0
5
10
15
20
25
30
35
40
H2B-RFP (DNA)
Cell mask (membrane)
Strong confinement (8x5 channel)
B
Naïve
p<0.0001
p=0.0229
p=0.0412
Asymmetry ratio
1.0
0.5
0.0
10*10
8*6
8*5
Channel section (µm*µm)
C
Exit
REX1-GFP Intensity (a.u.)
2,200
2,150
2,100
2,050
2,000
0
5
10
15
Time (h after division)

Figure S3

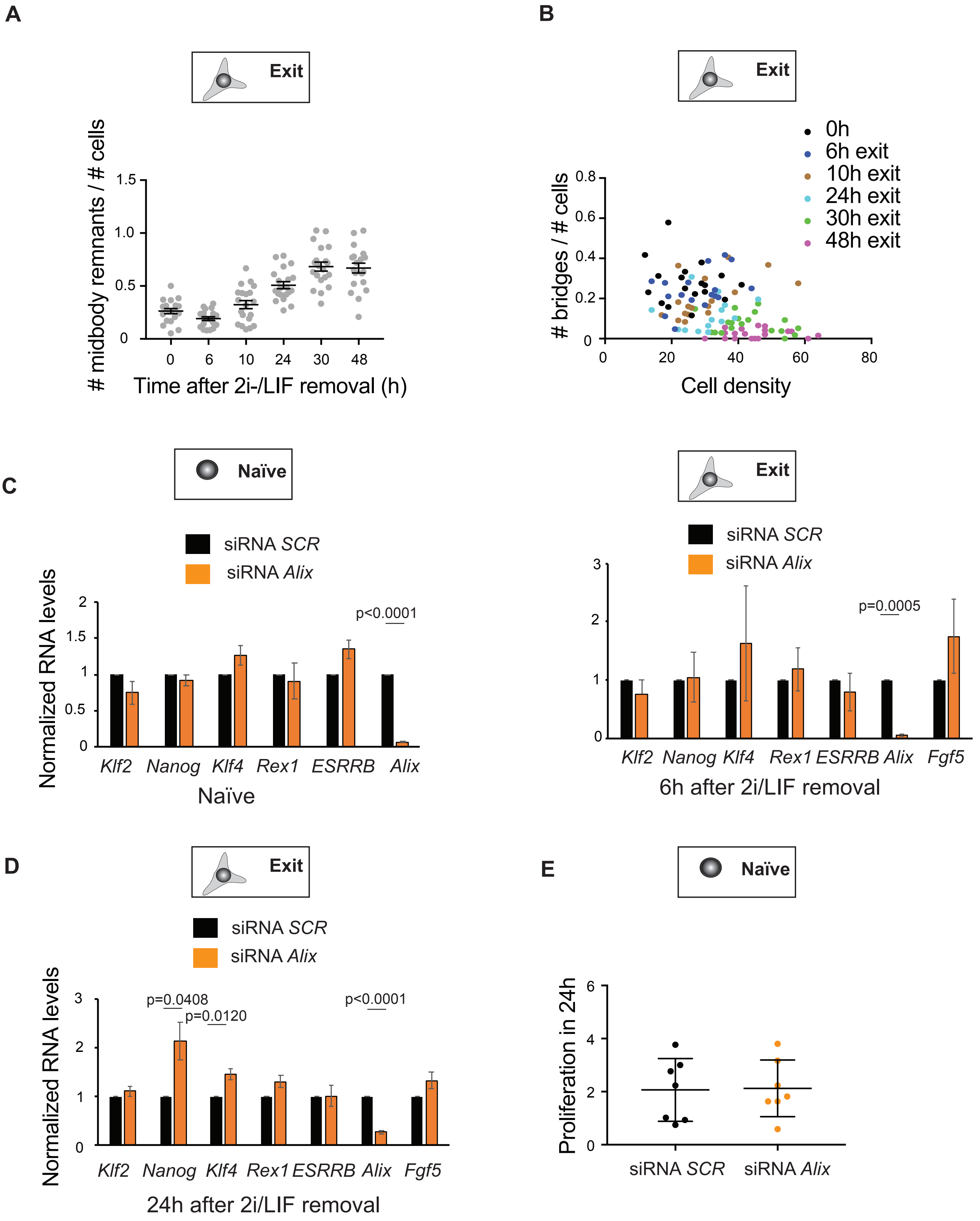
A
Exit
midbody remnants / # cells
Time after 2i-/LIF removal (h)
B
Exit
0h
6h exit
10h exit
24h exit
30h exit
48h exit
bridges / # cells
Cell density
C
Naïve
siRNA SCR
siRNA Alix
Normalized RNA levels
p<0.0001
Klf2 Nanog Klf4 Rex1 ESRRB Alix
Naïve
Exit
siRNA SCR
siRNA Alix
p=0.0005
Klf2 Nanog Klf4 Rex1 ESRRB Alix Fgf5
6h after 2i/LIF removal
D
Exit
siRNA SCR
siRNA Alix
Normalized RNA levels
p=0.0408
p=0.0120
p<0.0001
Klf2 Nanog Klf4 Rex1 ESRRB Alix Fgf5
24h after 2i/LIF removal
E
Naïve
Proliferation in 24h
siRNA SCR siRNA Alix

Figure S4

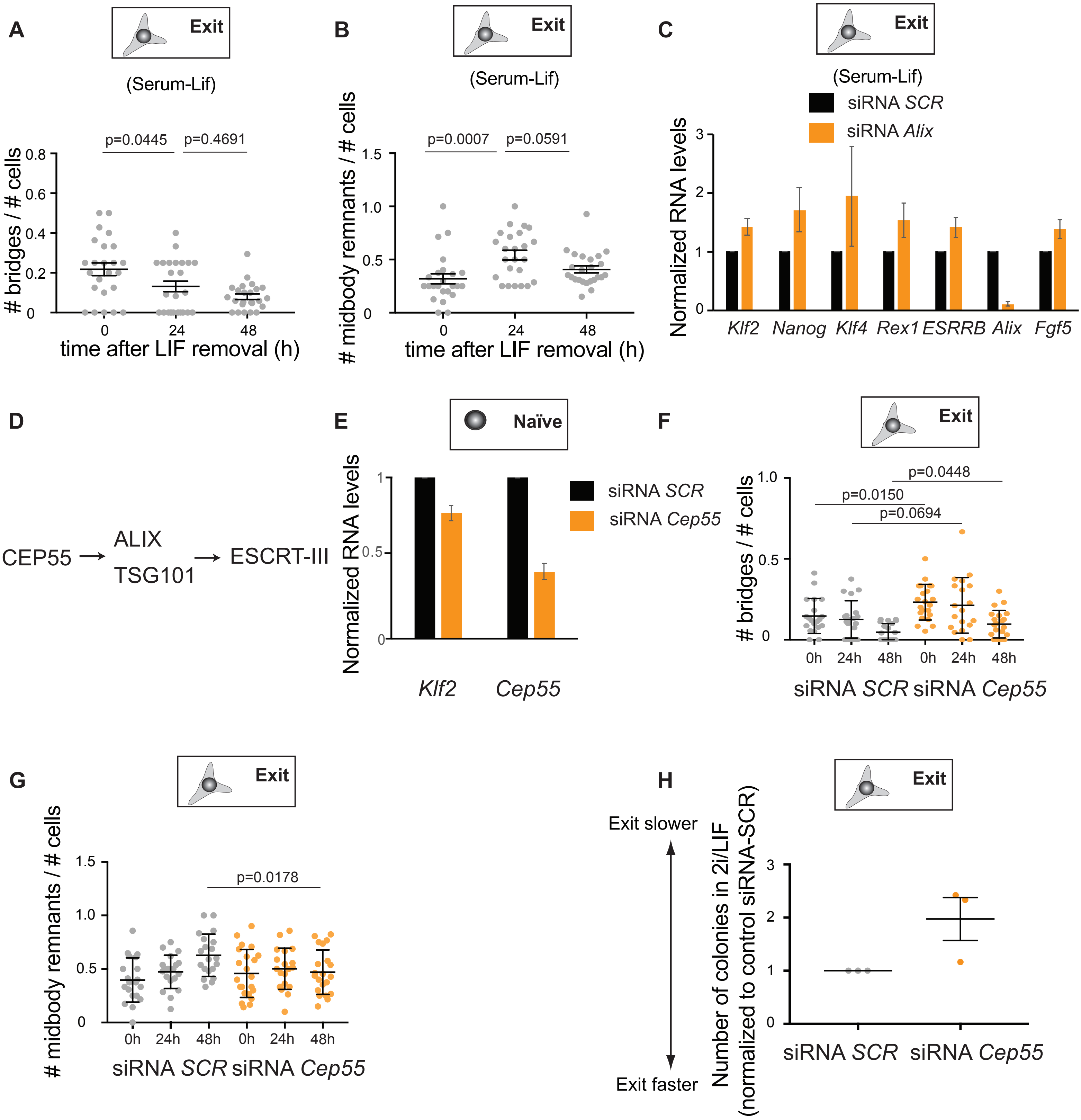
A
Exit
(Serum-Lif)
p=0.0445
p=0.4691
# bridges / # cells
time after LIF removal (h)
B
Exit
(Serum-Lif)
p=0.0007
p=0.0591
# midbody remnants / # cells
time after LIF removal (h)
C
Exit
(Serum-Lif)
siRNA SCR
siRNA Alix
Normalized RNA levels
Klf2 Nanog Klf4 Rex1 ESRRB Alix Fgf5
D
CEP55 → ALIX TSG101 → ESCRT-III
E
Naïve
Normalized RNA levels
siRNA SCR
siRNA Cep55
Klf2 Cep55
F
Exit
p=0.0448
p=0.0150
p=0.0694
# bridges / # cells
0h 24h 48h 0h 24h 48h
siRNA SCR siRNA Cep55
G
Exit
p=0.0178
# midbody remnants / # cells
0h 24h 48h 0h 24h 48h
siRNA SCR siRNA Cep55
H
Exit
Exit slower
Exit faster
Number of colonies in 2i/LIF (normalized to control siRNA-SCR)
siRNA SCR siRNA Cep55

Figure S5

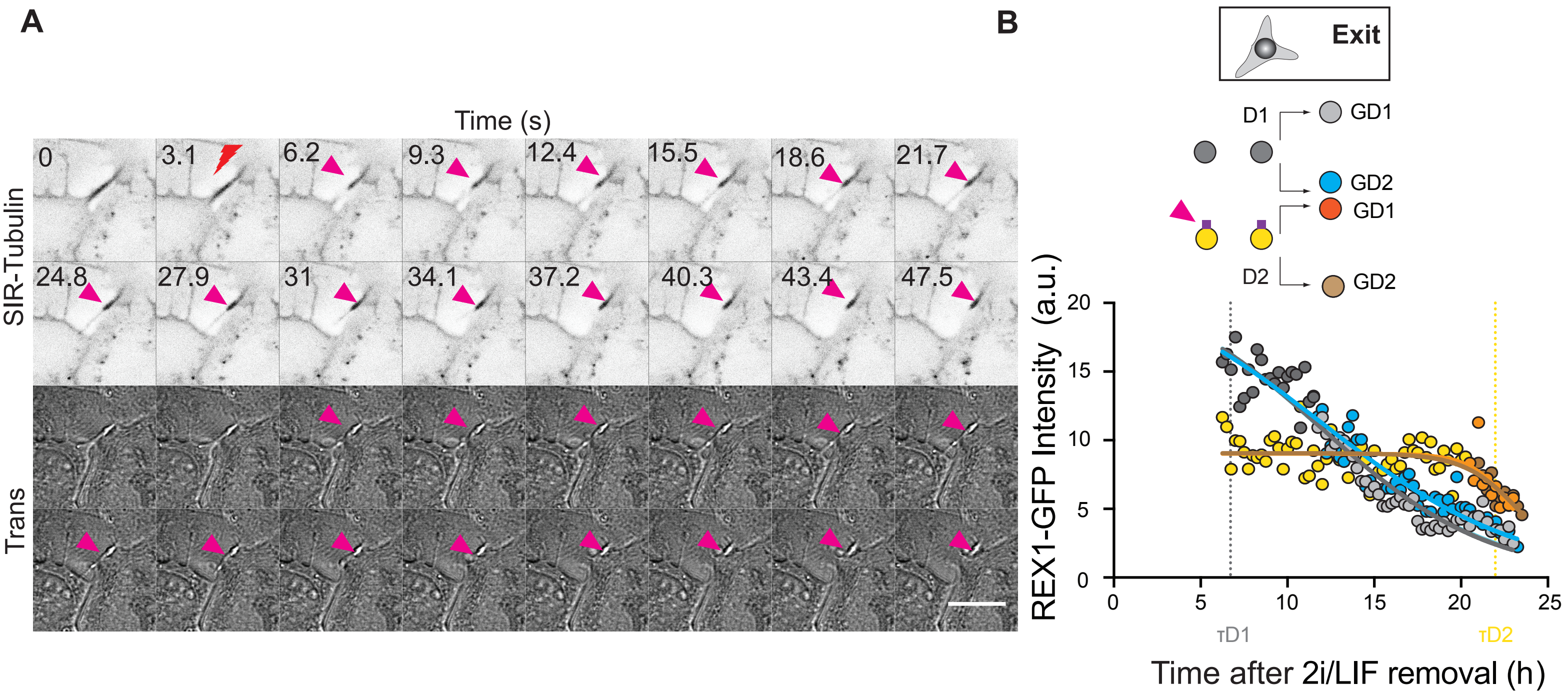
A
B
Time (s)
SIR-Tubulin
Trans
0
3.1
6.2
9.3
12.4
15.5
18.6
21.7
24.8
27.9
31
34.1
37.2
40.3
43.4
47.5
Exit
D1
GD1
GD2
GD1
D2
GD2
REX1-GFP Intensity (a.u.)
20
15
10
5
0
0
5
10
15
20
25
τD1
τD2
Time after 2i/LIF removal (h)

Figure S6
