# Supplementary material for: Abscission Couples Cell Division to Embryonic Stem Cell Fate
Source: Dev Cell. 2020 Oct 26;55(2):195–208.e5. doi: 10.1016/j.devcel.2020.09.001 (PMC7594744; doi:10.1016/j.devcel.2020.09.001)
Supplement: Document S2. Article plus Supplemental Information [file mmc8.pdf]

Developmental Cell

Article

Abscission Couples Cell Division to Embryonic Stem Cell Fate

Graphical Abstract

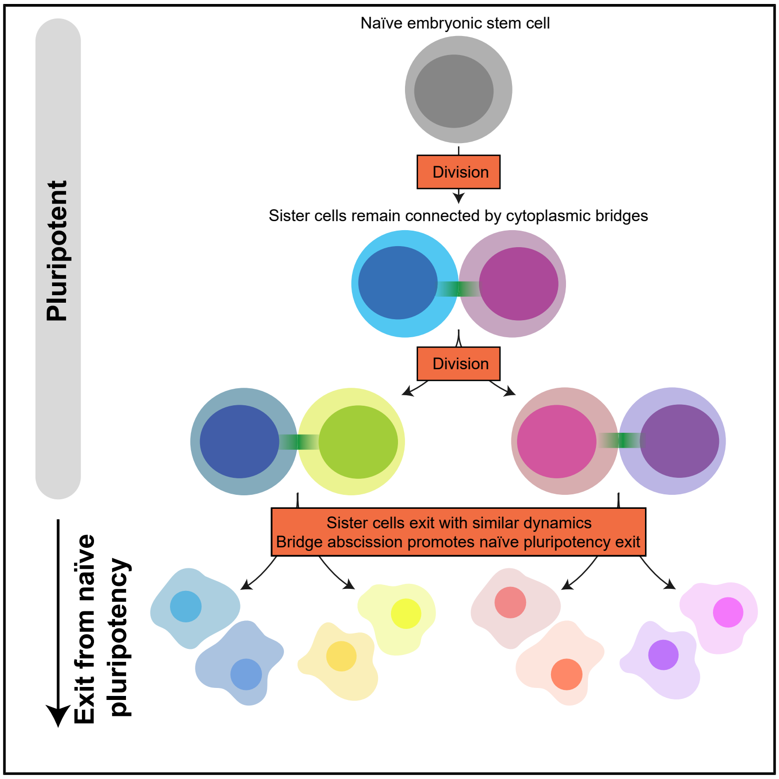

Highlights

- Mouse embryonic stem cells exit naive pluripotency after mitosis
- Naive embryonic stem cells display slow abscission and remain connected by bridges
- Cells exiting naive pluripotency display faster abscission
- Accelerating abscission facilitates exit from naive pluripotency

Authors

Agathe Chaigne, Céline Labouesse, Ian J. White, Meghan Agnew, Edouard Hannezo, Kevin J. Chalut, Ewa K. Paluch

Correspondence

a.chaigne@ucl.ac.uk (A.C.), ekp25@cam.ac.uk (E.K.P.)

In Brief

Cell division has been implicated in fate decisions in many stem cells. Chaigne et al. show that mouse embryonic stem cells exit naive pluripotency after cell division. Naive sister cells remain connected by cytoplasmic bridges after division, and bridge abscission promotes naive pluripotency exit.

Chaigne et al., 2020, Developmental Cell *55*, 195–208
October 26, 2020 © 2020 The Authors. Published by Elsevier Inc.
https://doi.org/10.1016/j.devcel.2020.09.001

CellPress

Developmental Cell

CellPress
OPEN ACCESS

Article

Abscission Couples Cell Division to Embryonic Stem Cell Fate

Agathe Chaigne,[1,*] Céline Labouesse,[2] Ian J. White,[1] Meghan Agnew,[1] Edouard Hannezo,[3] Kevin J. Chalut,[2] and Ewa K. Paluch[1,2,4,5,*]

[1]MRC Laboratory for Molecular Cell Biology, University College London, London WC1E 6BT, UK
[2]Wellcome/MRC Cambridge Stem Cell Institute, University of Cambridge, Cambridge CB2 0AW, UK
[3]Institute of Science and Technology Austria, Klosterneuburg 3400, Austria
[4]Department of Physiology, Development and Neuroscience, University of Cambridge, Cambridge CB2 3DY, UK
[5]Lead Contact
*Correspondence: a.chaigne@ucl.ac.uk (A.C.), ekp25@cam.ac.uk (E.K.P.)
https://doi.org/10.1016/j.devcel.2020.09.001

SUMMARY

Cell fate transitions are key to development and homeostasis. It is thus essential to understand the cellular mechanisms controlling fate transitions. Cell division has been implicated in fate decisions in many stem cell types, including neuronal and epithelial progenitors. In other stem cells, such as embryonic stem (ES) cells, the role of division remains unclear. Here, we show that exit from naive pluripotency in mouse ES cells generally occurs after a division. We further show that exit timing is strongly correlated between sister cells, which remain connected by cytoplasmic bridges long after division, and that bridge abscission progressively accelerates as cells exit naive pluripotency. Finally, interfering with abscission impairs naive pluripotency exit, and artificially inducing abscission accelerates it. Altogether, our data indicate that a switch in the division machinery leading to faster abscission regulates pluripotency exit. Our study identifies abscission as a key cellular process coupling cell division to fate transitions.

INTRODUCTION

During embryonic development and in adult tissue homeostasis, cell fate transitions allow the generation and maintenance of the diversity of cells constituting a functioning organism. The zygotic cell is totipotent, as it can give rise to all the embryonic and extra-embryonic tissues, and embryonic development relies on a series of precisely controlled fate transitions. In the adult organism, stem cells, for example in the gut or the skin, produce the cell types needed for tissue maintenance (Simons and Clevers, 2011). Understanding the cellular processes underlying fate transitions is thus of fundamental importance for development and physiology.

Cell division has been proposed to act as a switch during cellular fate transitions (Williams and Fuchs, 2013). A canonical example of mitotic control of cell fate is the first division of the *C. elegans* embryo, where cortical cues drive asymmetric spindle positioning, leading to asymmetries between daughter cells crucial for antero-posterior axis specification (Cowan and Hyman, 2004). In most oocytes, size asymmetry during meiosis is essential to ensure that the fertilized oocyte retains the reserves essential for embryo development, while the tiny polar body degenerates (Almonacid et al., 2014). In *Drosophila* and *C. elegans* neuroblasts, asymmetries in polarity determinant distribution correlate with size asymmetries between daughter cells, and in *C. elegans*, these size asymmetries have been proposed to directly control daughter cell fate after division (Cabernard and Doe, 2009; Ou et al., 2010). During embryonic development of the multicellular green alga *Volvox carteri*, cell size differences due to asymmetric divisions are also thought to dictate fate choice (Matt and Umen, 2016).

During early mammalian embryonic development, asymmetries at cell division can also lead to acquisition of distinct fates by the two daughter cells (Saini and Yamanaka, 2018). For instance, asymmetric inheritance of apical domains in the 8-to-16-cell mouse embryo leads to differences in cell mechanics, which in turn control positioning and fate acquisition (Maître et al., 2016). In culture, the importance of division for fate decisions of embryonic stem (ES) cells remains unclear. ES cells are derived from the early blastocyst and can indefinitely self-renew while retaining the capacity to give rise to all the cell types in the organism (Martello and Smith, 2014). Cell division has been linked to fate choice in human ES cells: when human ES cells exposed to primitive streak inducing signals divide, the two daughters cells often adopt different fates with one being resistant to differentiation (Brown et al., 2017). In mouse ES cells, artificially induced asymmetric division triggered by local application of beads coated with the signaling molecule Wnt3a leads to the daughter cell distal from the Wnt signal, expressing differentiation markers shortly after division (Habib et al., 2013). However, a number of studies suggest that in the absence of such external cues, lineage priming after naive pluripotency exit occurs in G1 phase (Liu et al., 2017; Pauklin and Vallier, 2013; Waisman et al., 2017). Nonetheless, overall inhibition of cell division

This is an open access article under the CC BY license (http://creativecommons.org/licenses/by/4.0/).

during naive pluripotency exit appears to affect transcriptional changes of some key pluripotency and differentiation markers, but not others (Waisman et al., 2017). Whether blocking cell division affects exit from naive pluripotency functionally has not been tested. Altogether, the importance of cell division for cell fate decisions in ES cells remains poorly understood.

Here, we investigate the role of cell division in exit from naive pluripotency using mouse ES cells as a model system. Using single-cell tracking, we show that naive pluripotency exit generally occurs after cell division. We then show that sister cells display highly correlated naive pluripotency exit timings, prompting us to test whether they remain connected even after division. Indeed, we find that abscission, the last stage of cell division, when sister cells become physically separated, is slow in naive ES cells, which remain connected by cytoplasmic bridges for a long time after division. Interestingly, abscission duration sharply decreases after naive pluripotency exit is triggered; our data suggest that this is due to a faster thinning of intercellular bridges in cells exiting the ES cell state, leading to faster recruitment of ESCRT-III components, which mediate the membrane scission itself. Finally, we show that interfering with abscission impairs, while inducing abscission by laser ablation speeds up naive pluripotency exit. Altogether, our findings unveil a rewiring of the division machinery, leading to faster abscission, as a key step in exit from naive pluripotency.

RESULTS

ES Cells Exit Naive Pluripotency after Mitosis

To investigate the role of cell division in exit from naive pluripotency, we first tested the effect of inhibiting cell division altogether. We used ES cells expressing a short half-life naive pluripotency reporter REX1-GFPd2 expressed from the endogenous REX1 locus (Kalkan et al., 2017; Strawbridge et al., 2020), since REX1 downregulation correlates with naive pluripotency exit (Kalkan et al., 2017; Mulas et al., 2017). ES cells were cultured in N2B27 medium supplemented with the MEK inhibitor PD0325901, the GSK-3 inhibitor CHIRON, and leukemia inhibitory factor (2i/LIF culture medium), and naive pluripotency exit was initiated by placing cells in N2B27 medium alone (differentiation medium hereafter) (Mulas et al., 2019). We blocked cell division with the CDK1 inhibitor RO-3306 and monitored REX1-GFPd2 (hereafter REX1-GFP) intensity after placing the cells in differentiation medium. While control cells showed a clear reduction of REX1-GFP intensity 40 h after inhibitors removal, consistent with previous reports (Kalkan et al., 2017; Mulas et al., 2017), cells that did not undergo cell division maintained higher REX1-GFP levels (Figures S1A and S1B). The efficiency of the division block was confirmed by comparing bulk proliferation of control and RO-3306-treated ES cells (Figure S1C). Furthermore, the RO-3306-treated cells were considerably larger than controls, as expected for cells blocked in G2 (Figures S1D and S1E). These data suggest that cell division is important for naive pluripotency exit, consistent with a previous study that had shown that downregulation of *Nanog*, another key naive pluripotency gene, was impaired in RO-3306-treated cells (Waisman et al., 2017).

To further test the importance of cell division, we asked how its timing relates to exit from the ES cell state. We used the onset of REX1-GFP downregulation as a readout of naive pluripotency exit timing, as *Rex1* is one of the last naive pluripotency genes to be downregulated in cells exiting the ES cell state (Kalkan et al., 2017). We first verified REX1 downregulation dynamics at the population level. We observed that after 25–40 h in differentiation media, all cells had downregulated REX1-GFP (Figure S1F; Video S1), consistent with previous reports (Kalkan et al., 2017). Furthermore, after 24 h in differentiation media, the cells had downregulated key genes of the naive pluripotency network (*Rex1*, *Klf2*, *Nanog*, and *Klf4*) and upregulated genes typical of early differentiation (*Fgf5* and *Otx2*) (Figure S1G). We then followed individual cells and their progeny to explore the correlation between cell division and REX1-GFP downregulation (Figures 1A–1D). The timing of REX1-downregulation was determined automatically, as the time of the first inflection of the curve in a sigmoidal fit to the time course of REX1-GFP intensity. Cell division appeared to correlate with the timing of REX1 downregulation (Figures 1A and 1B; Video S1). Interestingly, some of the cells did not downregulate REX1-GFP after the first division but did so after undergoing a second division (Figures 1C and 1D; Video S1). As a control, we verified that the levels of REX1-GFP in naive cells displayed little variability over the cell cycle, thus, confirming that the drop in REX1-GFP intensity after division in cells exiting the ES cell state was not the result of cell-cycle-linked changes in protein levels (Figure S1H). Taken together, we found that at the individual cell level, the time of naive pluripotency exit strongly correlated with the time of the latest division (Figure 1E). Finally, we confirmed that the correlation between time of REX1 downregulation and time of division was unlikely to be due to chance (Figure S1I and STAR Methods). Altogether, these results show that the timing of exit from naive pluripotency in ES cells correlates with cell division.

ES Cells Go through Most of a Cell Cycle and a Division before Exiting the ES Cell State

Since we observed that exit from naive pluripotency occurred shortly after a cell division, we hypothesized that placing cells in differentiation medium when they are about to enter mitosis could result in faster exit from the ES cell state. To test this hypothesis, we used FUCCI2a ES cells (Mort et al., 2014), which express different fluorescent markers in different phases of the cell cycle, and sorted cells in distinct cell-cycle phases. In order to functionally assess the effectiveness of exit from the ES cell state, we cultured cells in differentiation medium for 26 h and performed a clonogenicity assay (Figure 2A). In this assay, cells that have been cultured in differentiation medium for a determined period of time are placed back in 2i/LIF, where only naive pluripotent cells survive; a low number of cells surviving in the assay is thus a readout of efficient naive pluripotency exit (Figure 2A; Mulas et al., 2017). Interestingly, we found that cells placed in differentiation media at mitosis exit or while in G1 phase, exited naive pluripotency faster than control cells or cells synchronized in S/G2 phase, which are about to undergo cell division (Figure 2B).

To confirm these results at the single-cell level, we sorted wild-type ES cells by size, as small cells largely correspond to cells that just exited mitosis or are in G1 phase (Figure S2A). We then performed single-cell RNA sequencing on the small ("early cell-cycle") cells and on the unsorted population ("ungated") after 6 h in differentiation media, in order to capture the first transcriptional changes of naive pluripotency exit. We first verified that sorting

CellPress
OPEN ACCESS

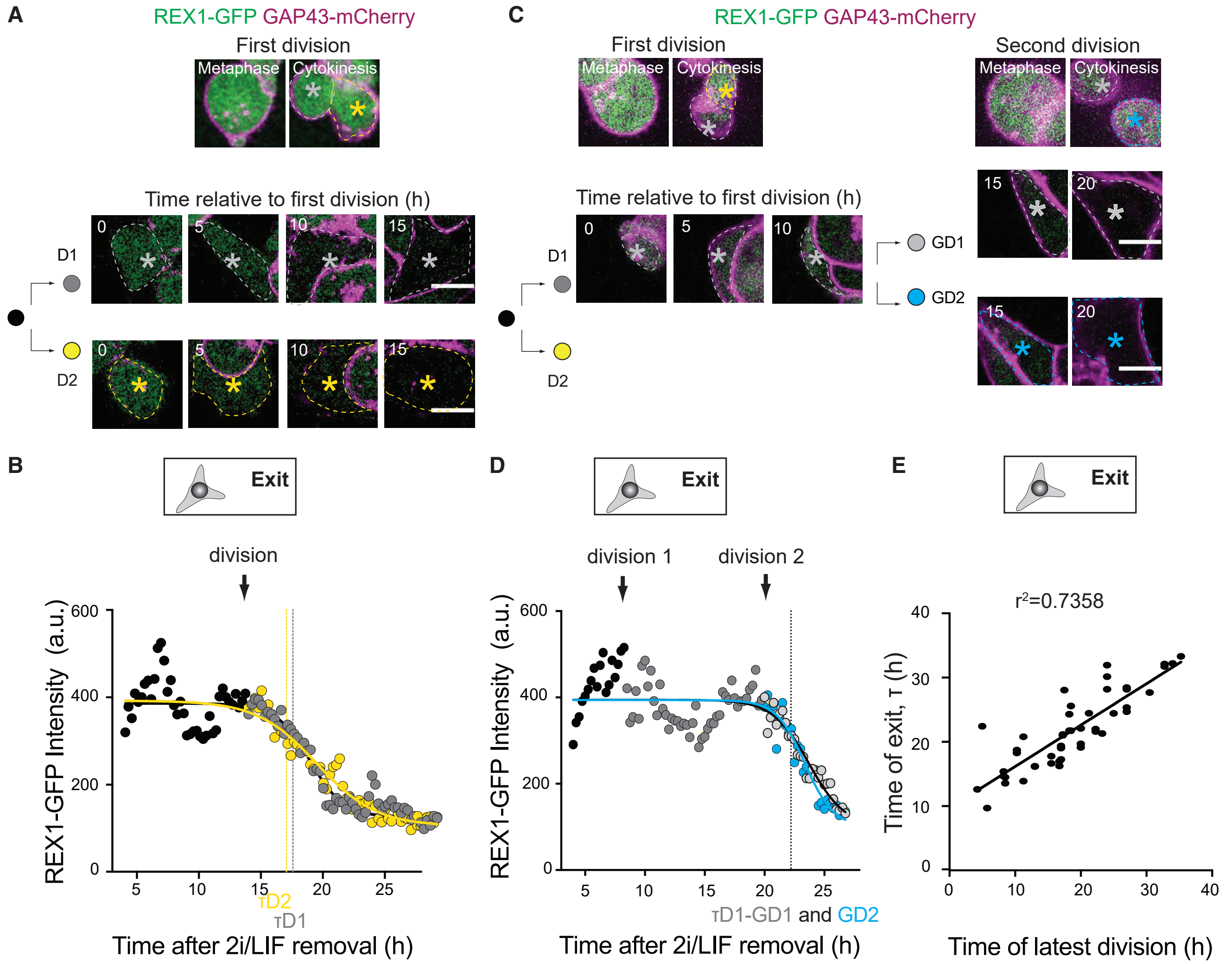

Figure 1. ES Cells Exit Naive Pluripotency after Mitosis

(A) Representative example of an ES cell expressing REX1-GFP (green) and GAP43-mCherry (magenta) undergoing one division before exiting naive pluripotency. Top, images of the cell division; bottom, time-lapse of the two daughter cells (D1 and D2) highlighted with stars in images at the top. 0 h: end of cytokinesis. A single Z plane around the center of the cell is shown. Scale bars: 10 μm.

(B) Plot of REX1-GFP mean intensity in the cells pictured in (A), as a function of time. 0 h: time of 2i/LIF removal. Black, mother cell; gray, daughter D1; yellow, daughter D2. Lines are sigmoidal decay fits; the time of REX1 downregulation (τ) is defined as the first inflection of the curve (see STAR Methods). The black arrow highlights the time of cell division.

(C) Representative example of an ES cells expressing REX1-GFP (green) and GAP43-mCherry (magenta) undergoing two divisions before exiting naive pluripotency. Top: images of the two cell divisions. Bottom: time-lapses of the daughter (D1 and D2) and granddaughter (GD1 and GD2) cells highlighted with stars in images at the top. 0 h: end of the first cytokinesis. A single Z plane around the center of the cell is shown. Scale bars: 10 μm.

(D) Plot of REX1-GFP mean intensity in the cells pictured in (C) as a function of time. 0 h: time of 2i/LIF removal. Black, mother cell; dark gray, daughter D1; blue, granddaughter GD1; light gray, granddaughter GD2. Lines are sigmoidal decay fits; the time of REX1 downregulation (τ) is defined as the first inflection of the curve (see STAR Methods). The black arrows highlight the times of the divisions.

(E) Scatter plot representing the time of REX1-GFP downregulation τ (readout of the time of naive pluripotency exit), as a function of the time of the latest division. The latest division is determined as the division that happens before or up to 2.5 h after (to account for experimental uncertainties in determining τ) the time of REX1-GFP downregulation. 0 h: time of 2i/LIF removal.

conserved the cell-cycle structure of the population and observed that 6 h after sorting and placing cells in differentiation media, the majority of cells from the initial "early cell-cycle" population were in G1 or S phase, whereas the ungated population comprised mostly S phase and G2/M cells (Figure S2B). We then compared the expression levels of key pluripotency genes and found that after 6 h in differentiation media, the cells for which naive pluripotency exit was triggered early in the cell cycle displayed overall stronger downregulation of pluripotency genes than the ungated population (Figure S2C). Furthermore, a cluster analysis separating the cells based on expression levels of two of the earliest genes downregulated during naive pluripotency exit, *Tfcp2l1* and *Tbx3* (Kalkan et al., 2017), indicated that "early cell-cycle" cells displayed a stronger downregulation of these early genes

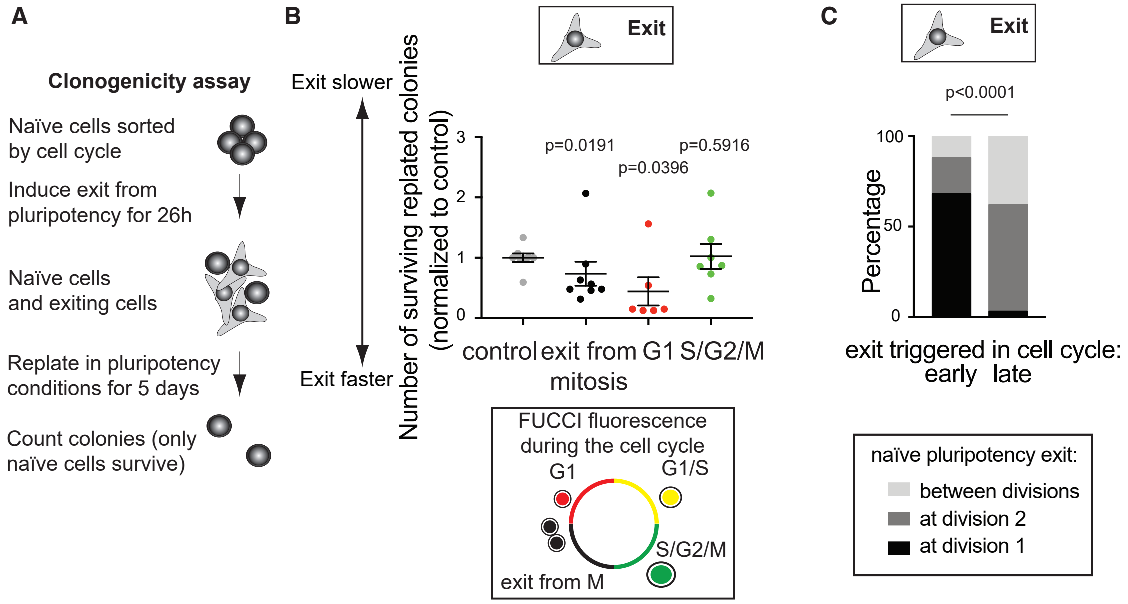

Figure 2. ES Cells Go through Most of a Cell Cycle before Exiting Naive Pluripotency

(A) Schematic of clonogenicity analysis assay (see STAR Methods).

(B) Dot plot representing the number of ES cell colonies surviving in a clonogenicity assay performed on cells synchronized in different phases of the cell cycle. FUCCI2a ES cells were synchronized by fluorescence-activated cell sorting (FACS), based on fluorescence (see schematic): cells in G1 express mCherry-Cdt1, cells in S, G2, and M phases express mVenus-hGeminin; cells at the G1/S transition are double positive and cells exiting mitosis are double negative. Control: ungated population. The mean and standard error of the mean are shown. N = 6 to 8 depending on the cell cycle stage.

(C) Percentage of cells downregulating REX1-GFP around the time of the first division (±4 h, black), around the time of the second division (±4 h, dark gray), or in between two divisions (light gray) for cells where naive pluripotency exit is triggered early in the cell cycle (the first division happens more than 12 h after 2i/LIF removal, left) or late in the cell cycle (the first division happens less than 12 h after 2i/LIF removal, right). N = 3, n = 200.

compared with the ungated population (Figure S2D). This is consistent with a previous report showing that when naive pluripotency exit is triggered in cells synchronized in G1 phase, downregulation of key naive pluripotency genes is initiated earlier than for cells where exit is induced later in the cell cycle (Waisman et al., 2017). Taken together, these results indicate that cells exit naive pluripotency faster when exit is triggered in cells that just finished mitosis (Figures 2A, 2B, and S2), yet mitosis itself appears to be important for loss of naive pluripotency (Figure 1).

To understand this, we further analyzed the correlation between cell division and REX1 downregulation (Figure 1). We separated the cell population into cells that divided shortly after 2i/LIF removal (less than 12 h), meaning 2i/LIF was removed late in the cell cycle, and cells that divided late after 2i/LIF removal, which means 2i/LIF was removed early in the cell cycle. Cells for which naive pluripotency exit was triggered early in the cell cycle mostly downregulated REX1-GFP at the first division, and cells for which exit was triggered late in the cell cycle predominantly downregulated REX1-GFP at the second division (Figure 2C). Altogether, these results suggest that ES cells go through most of a cell cycle and a division before exiting naive pluripotency.

ES Cells Present Strong Size Asymmetries between Daughter Cells at Cell Division, but Naive Pluripotency Exit Dynamics Are Insensitive to These Asymmetries

We then explored how division affects naive pluripotency exit. Since asymmetric divisions, in particular in size, are important for fate specification in a number of stem cell types (Brown et al., 2017; Cabernard and Doe, 2009; Cowan and Hyman, 2004; Ou et al., 2010), we asked if ES cells display cell division asymmetries. We monitored cell divisions in ES cells stably expressing H2B-RFP to label DNA (Cannon et al., 2015) and labeled with CellMask to mark the plasma membrane. Using 3D segmentation (Smith et al., 2017), we noticed that ES cells, in particular when dividing in colonies, displayed strong size asymmetries between daughter cells (Figures 3A and 3B). As a reference, HeLa cells, heavily derived cancer cells with great variability in chromosome count, which are thus not thought to control their size and division very precisely, divided much more symmetrically than mouse ES cells in colonies (Figure 3B). However, we did not observe significant differences in REX1-GFP intensity dynamics or downregulation timings between daughter cells, even when division was very asymmetric in size (Figures 3C and 3D). In fact, the timing of REX1 downregulation was strongly correlated between sister cells (Figure 3E) and the variance of REX1-GFP levels was very low between sisters (Figure 3F), consistent with a recent study analyzing REX1 dynamics in single cells during pluripotency exit (Strawbridge et al., 2020). These data indicate that sister cells exit the ES cell state in a highly correlated manner and suggest that size asymmetries at cell division do not influence the timing of naive pluripotency exit.

To directly test this, we induced strongly asymmetric divisions by confining ES cells in microchannels, as confinement has been shown to induce asymmetries at cell division in other cell types (Cadart et al., 2014, 2018). Confinement reliably induced division

CellPress
OPEN ACCESS

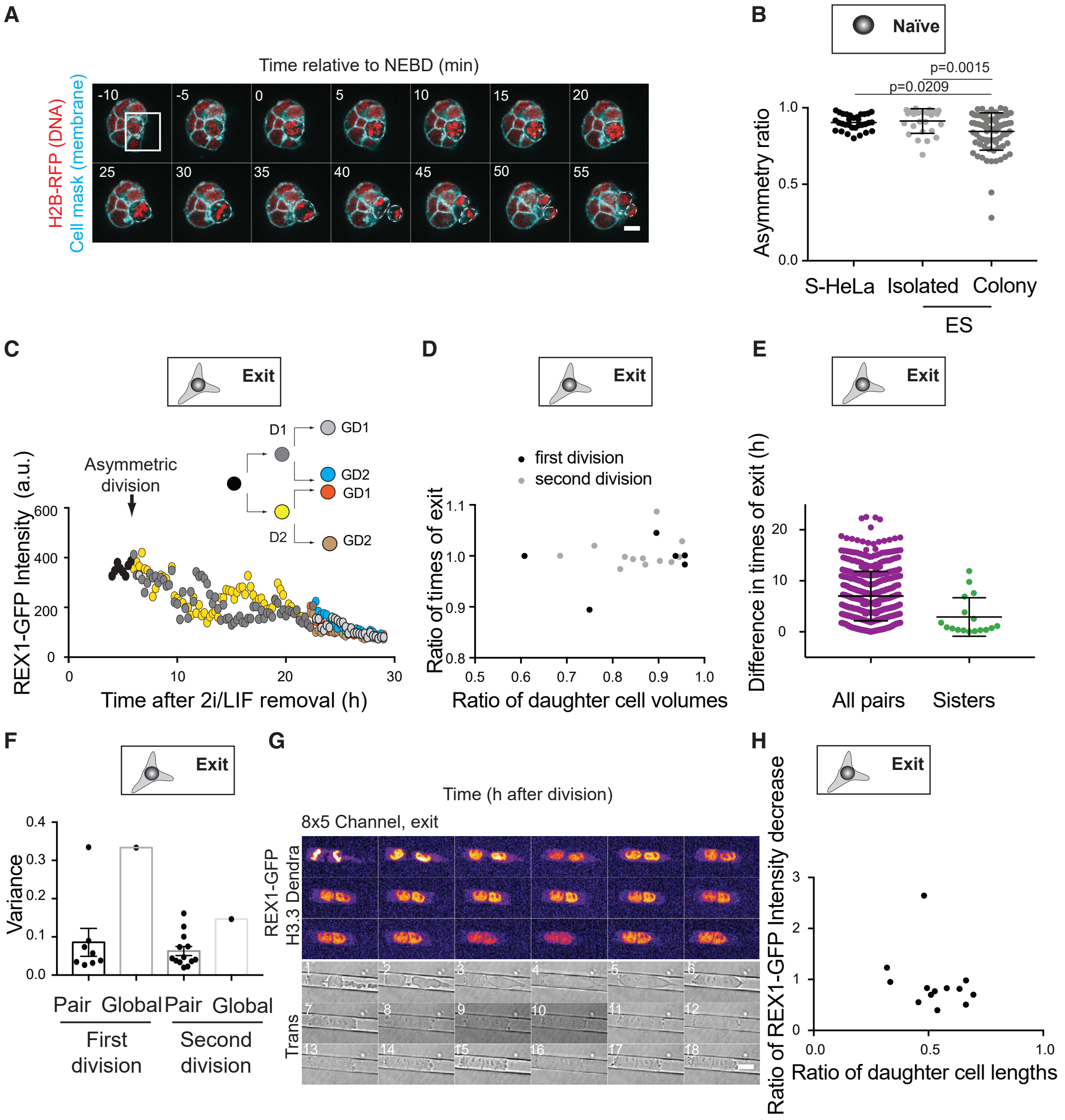

Figure 3. Daughter Cells Display Correlated Naive Pluripotency Exit Dynamics

(A) Representative time-lapse of a colony of naive ES cells expressing H2B-RFP (red) and labeled with CellMask^{TM} deep red (cyan) with one cell dividing asymmetrically (white box). Time in min; 0 min: time of nuclear envelope breakdown (NEBD). A single Z plane is shown. Scale bar: 10 μm.

(B) Dot plot representing the ratio between the volumes of the smaller and bigger daughter cell (asymmetry ratio) 15 min after cytokinesis for suspension HeLa cells (S-HeLa, used as a reference, black), single ES cells (“isolated,” light gray) and ES cells dividing in the colony (“colony,” dark gray). Mean and standard deviation are plotted. N = 3.

(C) Example plot of REX1-GFP mean intensity for cells exiting naive pluripotency after a very asymmetric division (ratio of volumes: 0.59) as a function of time. 0 h: time of 2i/LIF removal.

(D) Plot showing the ratio between the times of REX1-GFP downregulation after 2i/LIF removal (times of exit) for sister cells exiting naive pluripotency at the first (black) or second (gray) division, as a function of the ratio of the volumes of the sister cells. N = 3, n = 18 pairs of sisters.

(E) Dot plot showing the absolute difference in naive pluripotency exit time for pairs of cells chosen at random (left) and pairs of sister cells (right). Mean and standard deviation are plotted. N = 3.

(F) Dot plot showing the variance (averaged over time) in intensity of the REX1-GFP signal, for cells exiting naive pluripotency at the first division (left) or the second division (right), comparing variance for pairs of sister cells (“pair”) and the global average variance of all cells (“global,” see STAR Methods for details). N = 3.

(G) Time-lapse of an ES cell expressing Dendra2-H3.3-N-14 (H3.3 Dendra, to visualise DNA) and REX1-GFP (fire, upper panel) dividing in an 8 × 5 μm channel in N2B27. The transmitted light channel for monitoring cell length is shown in the bottom panel. One picture is shown every 1 h. 0, anaphase. One Z plane is shown. Scale bar: 10 μm.

(H) Plot showing the ratio of the decrease in REX1-GFP cytoplasmic intensity as a function of the ratio of daughter cell lengths 6 h after cell division in the channels. N = 6.

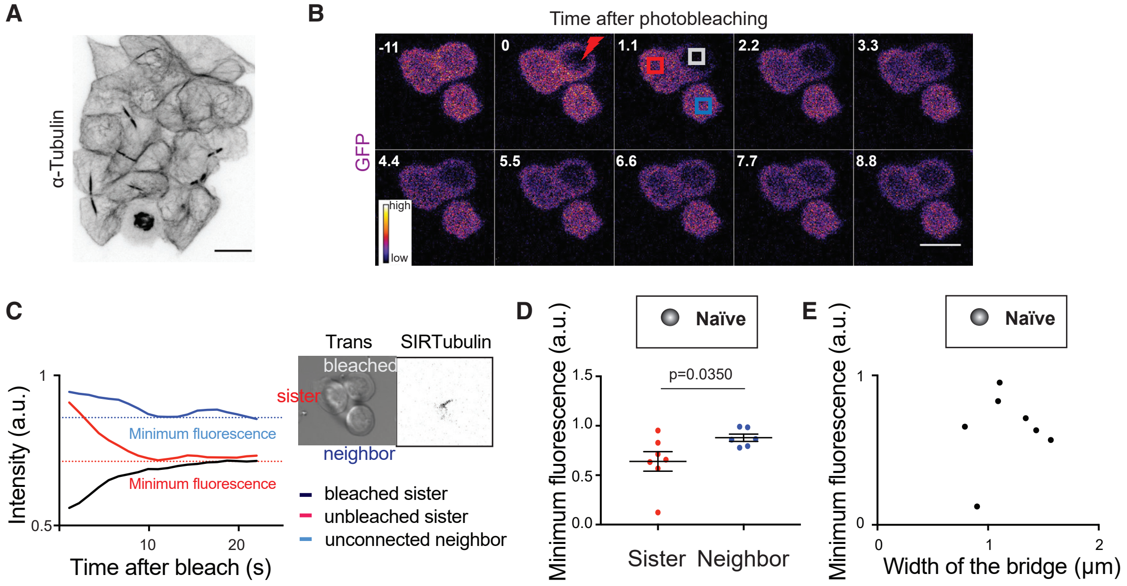

Figure 4. Naive ES Cells Remain Connected after Division

(A) Representative confocal image of a naive ES cell colony stained for α-tubulin (black, inverted contrast). A maximum Z projection is shown. Scale bar: 10 μm.

(B) Representative time-lapse of a fluorescence recovery after photobleaching (FRAP) experiment in ES cells expressing cytoplasmic GFP, the two cells at the top are sister cells. GFP intensity levels are displayed. Photobleaching is performed at 0 s in the sister cell on the right (red lightning bolt). GFP intensity is then monitored in the bleached cell (dark gray box), the sister cell (red box) and an unconnected neighbor (blue box). One Z plane is shown, time in seconds, scale bar: 10 μm.

(C) Left panel: plot showing the mean GFP intensity over time (smoothed over a 4 point, ~4.5 s, window) in the boxes in the cells depicted in (B) Dotted lines highlight minimum fluorescence levels for the two unbleached cells. Right panel: transmitted light image and fluorescent Z projection (inverted contrast) of the 3 cells displayed in (B) and labeled with SIR-tubulin prior to the FRAP experiment, highlighting the tubulin bridge connecting the two cells at the top.

(D) Dot plot showing the minimum GFP levels in the sister cell of the ES cell where GFP was bleached (red) and for an unconnected neighboring cell at a similar distance (blue). Mean and standard error of the mean are plotted. N = 3.

(E) Dot plot showing the minimum GFP levels following photobleaching in one cell with a bridge, in its unbleached connected sister cell (data from D), as a function of the width of the bridge connecting the two cells. N = 3.

asymmetries in ES cells (Figures S3A and S3B; Video S2). We found that in the hours following cell division in microchannels, REX1-GFP levels displayed similar levels in the two daughter cells (Figures 3G, 3H, and S3C), and no correlation was observed between the size ratio of the daughter cells and the ratio of REX1-GFP intensity decrease in the two daughter cells 6 h after cell division (Figure 3H). In conclusion, size asymmetries between daughter cells at cell division do not appear to influence the timing of naive pluripotency exit.

Sister Cells Remain Connected after Division in ES Cells

The strong correlation in REX1 downregulation dynamics between daughter, and in some cases granddaughter cells (Figures 3E and 3F) led us to ask whether daughter cells might remain connected after division. We thus imaged microtubules and observed that naive ES cell colonies displayed a high number of tubulin bridges, remnants of mitotic spindles, still connecting daughter cells (Figure 4A). We then asked if sister cells connected by a bridge could still exchange cytoplasmic material. We expressed cytoplasmic GFP and used photobleaching to abruptly decrease cytoplasmic intensity. We found that photobleaching in one sister cell led to a decrease in cytoplasmic GFP intensity in the other sister, but not in a nearby unconnected cell positioned at a similar distance, indicating exchange of cytoplasmic material between the two connected sister cells (Figures 4B–4D; Video S3). Finally, we observed no correlation between bridge width and the apparent amount of transfer of cytoplasmic GFP (using the amplitude of fluorescence decay in the sister cell as a proxy) between the two sister cells (Figure 4E). As bridge width decreases over time (Mierzwa and Gerlich, 2014), this suggests that the daughter cell cytoplasms remainconnected even late after cell division. Taken together, these results suggest that abscission is slow in ES cells and that sister ES cells remain physically connected and exchange cytoplasmic material after cell division.

Abscission Duration Decreases during Exit from Naive Pluripotency

Since sister cells remain physically connected by intercellular bridges after division and appear to exit the ES cell state with similar dynamics, we hypothesized that abscission, the last step of cell division when sister cells physically separate, could be important for naive pluripotency exit. To explore changes in abscission during naive pluripotency exit, we immuno-stained tubulin and the midbody marker Citron Rho-interacting kinase (CRIK) (Hu et al., 2012) to identify abscission bridges and midbodies in ES cells and cells at various stages of naive pluripotency exit. All bridges were found to display CRIK foci, but some CRIK foci were not associated with bridges, suggesting they mark midbody remnants (Figure 5A). We found that the

CellPress
OPEN ACCESS

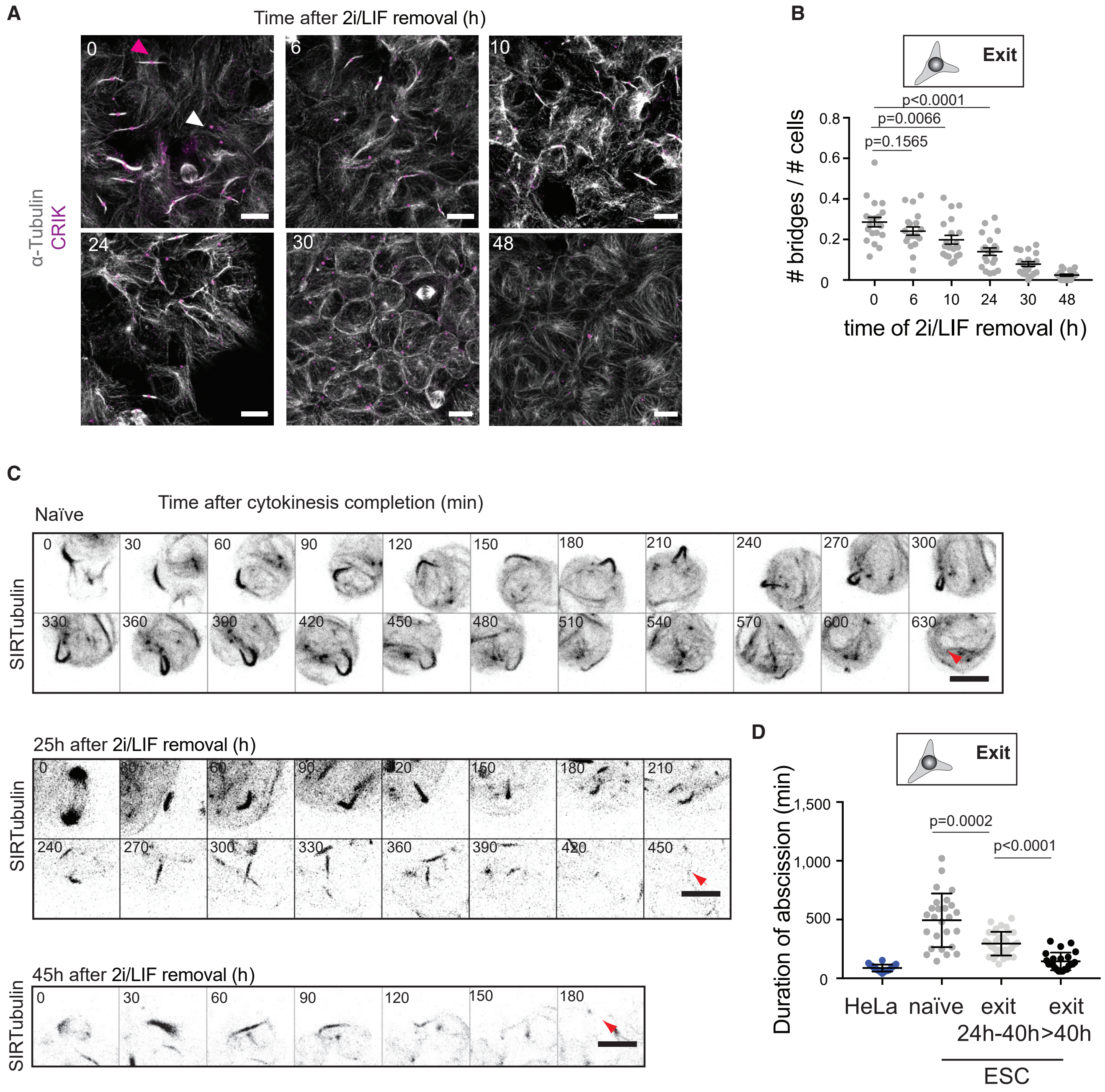

Figure 5. Abscission Duration Decreases during Naive Pluripotency Exit

(A) Representative confocal images of cells at different stages of naive pluripotency exit and stained for α-tubulin (white) and CRIK (magenta). Pink arrowhead, example of a bridge with a CRIK spot; white arrowhead, example an isolated CRIK spot, suggesting a midbody remnant. Cells are cultured on laminin to facilitate the visualization of the bridges. Scale bars: 10 μm.

(B) Dot plot showing the fraction of cells with bridges (number of bridges divided by number of cells in a given analysis frame) in H2B-RFP ES cells and during naive pluripotency exit on laminin. Mean and standard error of the mean are shown. N = 2.

(C) Representative time-lapses of a colony of ES cells expressing H2B-RFP and labeled with SIR-tubulin (black, inverted contrast, maximum Z projection across the colony is shown). Time in min. 0 min: end of cytokinesis. Top, naive cells; Middle and bottom, cells 25 and 45 h after induction of naive pluripotency exit, respectively. Red arrows: abscission. Scale bars: 10 μm.

(D) Dot plot showing the duration of abscission for HeLa cells expressing tubulin-GFP dividing on elongated line micropatterns to standardize cell shape (blue) and for naive ES cells and cells exiting naive pluripotency labeled with SIR-tubulin (gray and black dots). Mean and standard error of the mean are shown. N = 3.

fraction of cells with bridges decreased (Figures 5A and 5B), while the density of midbody remnants increased (Figure S4A) during naive pluripotency exit, suggesting that bridge abscission may progressively become faster. To further characterize abscission dynamics, we acquired time-lapse videos of cells treated with low doses of SIR-tubulin (Lukinavičius et al.,

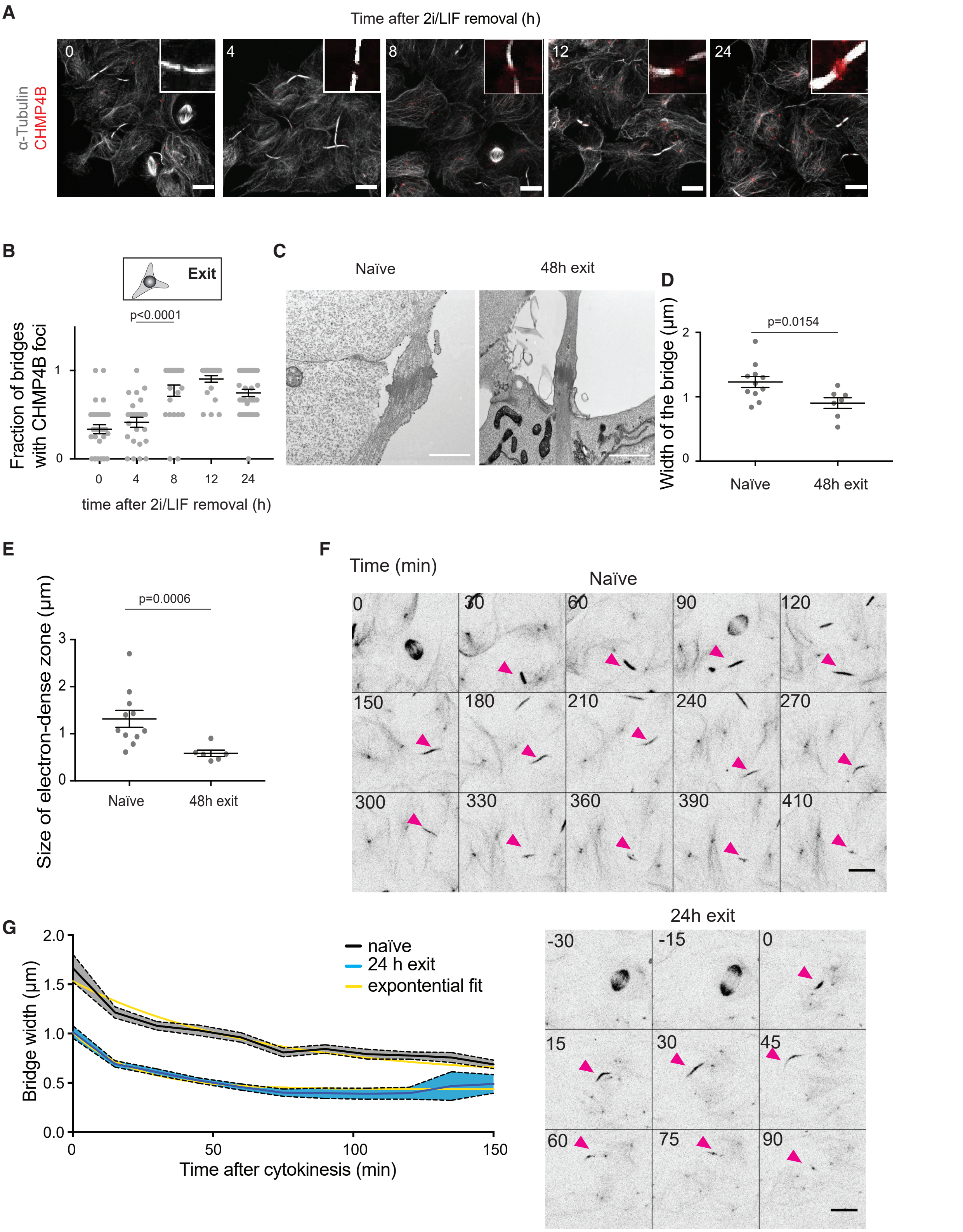

Figure 6. Bridge Thinning and CHMP4B Recruitment Accompany Shortening of Abscission Duration during Naive Pluripotency Exit

(A) Representative confocal images showing cells stained for α-tubulin (white) and CHMP4B (red) during naive pluripotency exit. Inset: zoom of representative bridges. Scale bars: 10 μm.

(legend continued on next page)

2014), a live marker of tubulin, and measured the duration of abscission (time between cytokinesis and bridge abscission, Figures 5C and 5D; Video S4). Of note, we used the time of microtubule bridge dissolution as a readout of abscission timing, which might precede the time of actual membrane severing (Guizetti et al., 2011). We found that naive ES cells maintained tubulin bridges much longer than HeLa cells, used as reference (1.5 ± 0.5 h in HeLa cells, consistent with Guizetti et al. [2011] versus 8.2 ± 3.8 h in naive ES cells; Figures 5C and 5D), further indicating that abscission takes a long time in ES cells. Abscission duration then decreased during exit from naive pluripotency (Figures 5C and 5D). Together, these results indicate that abscission is slow in naive ES cells, and that abscission duration decreases during naive pluripotency exit.

CHMP4B Recruitment and Accelerated Bridge Thinning Accompany Exit from Naive Pluripotency

We then explored the mechanisms underlying the change in abscission dynamics during naive pluripotency exit. Previous work in fibroblasts showed that abscission duration decreases with increasing cell density (Lafaurie-Janvore et al., 2013). Significantly changing cell density is challenging in ES cells, which spontaneously form aggregates. Nonetheless, we observed a slight inverse correlation between the fraction of cells with bridges and naturally occurring cell densities (Figure S4B), suggesting that increasing cell density might accelerate abscission. We then asked whether the faster abscission dynamics as cells exit the ES cell state could result from molecular changes in bridge composition. Notably, the expression levels of key known abscission regulators do not extensively change during naive pluripotency exit (Table S1, data from Kalkan et al. [2017] and Yang et al. [2019]). We thus probed changes in localization, focusing on the ECRT-III protein CHMP4B. CHMP4B performs the last step of abscission by polymerizing into circular filaments that are thought to cut the bridge (Chiaruttini et al., 2015; Mierzwa et al., 2017) and as such can be used as a readout of bridge maturity. We found that only 33% of the bridges in naive ES cells displayed CHMP4B foci (Figures 6A and 6B). The fraction of bridges with CHMP4B foci increased during pluripotency exit (Figures 6A and 6B), with a particularly strong increase between 4 and 8 h after triggering exit from naive pluripotency (Figure 6B).

To investigate what could modulate CHM4B recruitment, we asked whether bridge structure changed between ES cells and cells exiting the ES cell state. Indeed, abscission relies on two consecutive steps: first, an actin-dependent constriction that corresponds to cytokinesis, leading to bridge and midbody formation; second, a bridge maturation phase, during which bridge thinning precedes ESCRT-III components recruitment (reviewed in Mierzwa and Gerlich, 2014). We thus analyzed bridge structure using electron microscopy (Figure 6C). We observed that bridge width and the size of the central electron dense zone were significantly smaller in cells exiting the ES cell state than in naive cells (Figures 6D and 6E), suggesting that the bridge maturation phase, during which the bridge narrows, might be faster. We then analyzed the dynamics of bridge thinning (Figures 6F and 6G) and found that when cells exit naive pluripotency, the bridge gets thinner faster than in naive cells (Figure 6G). Together, these results strongly suggest that abscission bridges are structurally different between naive cells and cells exiting the ES cell state and that the bridge-thinning phase is accelerated when cells exit naive pluripotency, leading to a faster recruitment of ESCRT-III components (Figures 6A and 6B), which in turn drive the final stage of abscission.

Abscission Gates Exit from Naive Pluripotency

Finally, we asked whether interfering with abscission affects naive pluripotency exit. First, we depleted ALIX, which regulates the recruitment of ESCRT-III components to the bridge. ALIX depletion is expected to interfere with abscission (Carlton and Martin-Serrano, 2007; Morita et al., 2007), without significantly affecting intercellular trafficking processes (Adell and Teis, 2011). In naive ES cells, siRNA against *Alix* did not affect the expression of key pluripotency markers but efficiently decreased ALIX expression (Figure S4C). ALIX depletion impaired the decrease in bridge density after induction of naive pluripotency exit, suggesting that it effectively targets abscission (Figures 7A and 7B). We then performed a clonogenicity assay and found that ALIX depletion impaired exit from naive pluripotency (Figure 7C). We further found that 24 h after triggering naive pluripotency exit, the pluripotency genes *Nanog* and *Klf4* maintained high expression levels in ALIX-depleted cells compared with controls (Figure S4D). We verified that knocking down *Alix* did not impair cell proliferation (Figure S4E). We also verified that the effect of abscission on naive pluripotency exit was not due to the specific culture conditions and repeated these experiments using an alternative pluripotency-promoting culture medium (serum/LIF). We found that when ES cells exited naive pluripotency from serum/LIF, they also presented a decrease in bridge density (Figure S5A), an increase in midbody density (Figure S5B), and ALIX siRNA also impaired naive pluripotency exit (Figure S5C). Finally, we depleted the midbody protein CEP55, which is responsible for targeting ALIX and ESCRT-I

(B) Dot plot showing the fraction of bridges displaying a CHMP4B spot near the bridge center in cell colonies during naive pluripotency exit on laminin. Mean and standard error of the mean are shown. N = 2.

(C) Representative electron microscopy images of bridges connecting two naive cells (left) and two cells after 48 h in differentiation medium (right). Scale bars: 1 μm.

(D) Dot plot showing bridge width measured from electron microscopy images of ES cells and cells exiting naive pluripotency. Mean and standard error of the mean are plotted. N = 2.

(E) Dot plot showing the size of the electron dense midzone of the bridge, corresponding to the midbody, measured from electron microscopy images of ES cells and cells exiting naive pluripotency. Mean and standard error of the mean are plotted. N = 2.

(F) Representative time-lapses of tubulin bridges in ES cells (top) or cells after 24 h in differentiation medium (bottom) labeled with SIR-tubulin (black, inverted contrast), Z projections are shown. Time in min. The pink arrowheads point to the bridges. Scale bars: 10 μm.

(G) Time course of bridge width after cytokinesis for naive cells (black) and cells after 24 h in differentiation medium (blue). 0 min: cytokinesis. Mean and standard error of the mean are shown. N = 2, n = 22 for naive cells and 55 for cells after 24 h in differentiation medium. Exponential fits (yellow) show a faster decrease in bridge width in exiting cells (characteristic times: 45 min in naive cells versus 15 min in exiting cells, $p < 0.0001$).

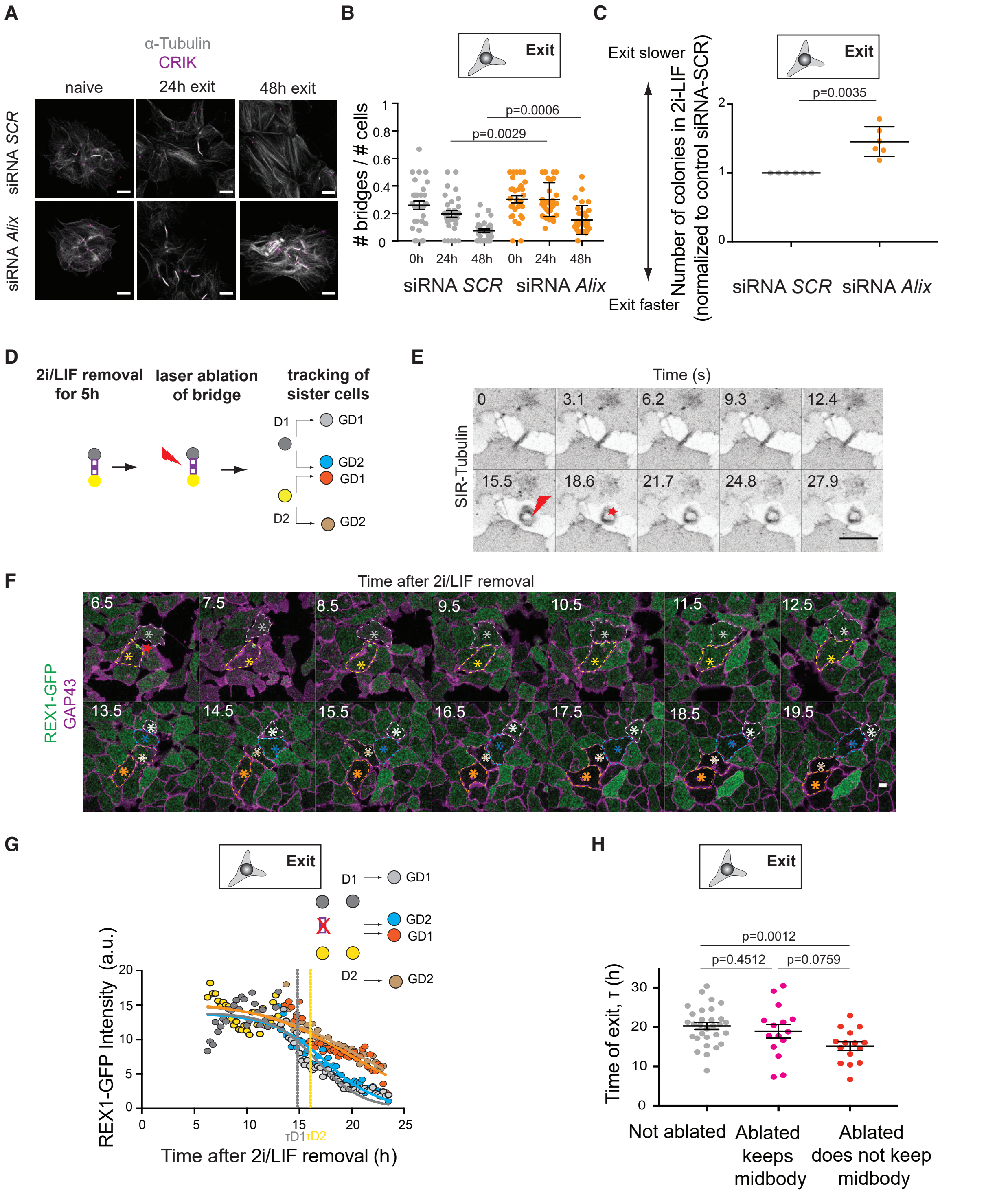

Figure 7. Abscission Regulates Exit from Naive Pluripotency

(A) Confocal images of H2B-RFP ES cells treated with scrambled siRNA (SCR, top) or siRNA against *Alix* (bottom) for 24 h in 2i/LIF, then plated on laminin in 2i/LIF or N2B27 (24 and 48 h exit), and stained for α-tubulin (white) and CRIK (magenta). A maximum Z projection over the volume of the colony is shown. Scale bars: 10 μm.

(B) Dot plot showing the fraction of cells with bridges (number of bridges divided by number of cells in a given analysis frame) in ES colonies pre-treated with siRNA *Scrambled* (*SCR*, gray) or *Alix* (orange) for 24 h in 2i/LIF, during naive pluripotency exit on laminin. Mean and standard error of the mean are shown. N = 2.

(C) Dot plot representing the number of colonies surviving in a clonogenicity assay (see Figure 2A) for ES cells treated with siRNA *Scrambled* (*SCR*, gray) or *Alix* (orange) for 24 h in 2i/LIF, then placed in differentiation media for 24 h. The mean and standard deviation are shown. N = 6.

(legend continued on next page)

components to the bridge (Figure S5D). Knockdown of *Cep55* was sufficient to maintain a high number of bridges during exit from naive pluripotency and a low midbody remnant density (Figures S5E–S5G). Furthermore, CEP55 depletion impaired exit from naive pluripotency (Figure S5H). Altogether, these results suggest that a quicker resorption of intercellular bridges after removal of pluripotency-promoting media promotes exit from naive pluripotency.

To directly test whether bridge resolution promotes naive pluripotency exit, we disrupted bridges by laser ablation 5 h after 2i/LIF removal (Figures 7D and 7E; Video S5). Laser ablation did not impair cell viability as ablated cells continued to divide normally (Figure 7F; Video S6). Strikingly, in cases where the midbody was not destroyed, the cells that retained the midbody after ablation displayed a timing of REX1 downregulation comparable to non-ablated controls, whereas cells for which ablation led to loss of midbody because it was either destroyed (Figures 7F and 7G; Video S5, left) or retained by the other cell (Video S5, right), displayed significantly faster REX1 downregulation dynamics (Figures 7H, S6A, and S6B). Altogether, these results suggest that triggering premature midbody release speeds up naive pluripotency exit.

DISCUSSION

Using a combination of functional assays and single-cell tracking, we have shown that naive pluripotency exit, as assessed by the timing of REX1 downregulation (Kalkan et al., 2017), occurs after cell division. When exit is induced early in the cell cycle, cells downregulate REX1 after the first division, whereas when exit is induced later in the cell cycle, cells generally undergo two divisions before REX1 downregulation (Figures 1 and 2). We conclude that cells need to go through most of a cell cycle and a division to effectively exit the ES cell state. Linking fate decisions to the cell cycle is a common feature in numerous types of multipotent cells. For instance, in human pluripotent stem cells, G1 phase has been proposed to act as a "window of opportunity" for dissolution of the pluripotency state (Pauklin and Vallier, 2013; Gonzales et al., 2015); subsequent lineage priming also generally occurs in G1 phase but could be affected by mitotic bookmarking, which maintains epigenetic marks during mitosis allowing for rapid gene activation in G1 phase (reviewed in Soufi and Dalton [2016]). In mouse ES cells, G1 phase is often considered a key stage for triggering pluripotency exit, because it allows for differentiation signals to subsequently rewire gene expression during DNA replication in S phase (Waisman et al., 2017), and because G1 Cyclin-CDKs have been shown to directly stabilize the core pluripotency network (Liu et al., 2017). However, so far, most studies have focused on exploring how transcriptional network rewiring is affected by cell-cycle signaling. In contrast, whether the cellular processes associated with the cell cycle could also affect fate transitions has received little attention.

Our study identifies the last cellular process of cell division, abscission, which can happen in G1 phase or later (Gershony et al., 2014), as a permissive cue for naive pluripotency exit. We found that abscission is slow in mouse ES cells, leading to cells remaining connected by cytoplasmic bridges for a long time after cell division, and that abscission accelerates during naive pluripotency exit. This is in line with previous observations that enhanced midbody release, and thus enhanced abscission, accompany cell differentiation in a number of cell types (Ettinger et al., 2011). Here, we show that maintaining bridges impairs naive pluripotency exit (Figures 7A–7C and S5D–S5H), while premature abscission and midbody release accelerates REX1-GFP downregulation (Figures 7D–7H). Interestingly, a link between the presence of stable cytokinetic bridges and cell potency can be highlighted in various species and at various developmental stages, including in mouse, frog, insect, and ctenophore germ cells, in cnidaria interstitial cells, and in the early mouse embryo (Alié et al., 2011; Coggins, 1973; David, 2012; Matias et al., 2015; Pepling and Spradling, 1998; Pepling et al., 1999; Zenker et al., 2017). Early mouse embryos have been shown to retain tubulin bridges throughout interphase from the 2-cell stage up to the blastocyst stage, and these interphase tubulin bridges have been proposed to act as a platform for E-cadherin transport toward cell-cell junctions (Zenker et al., 2017). E-cadherin has been implicated in pluripotency maintenance (Soncin et al., 2009); thus, long-lived tubulin bridges could help maintain pluripotency in ES cells by ensuring E-cadherin targeting to cell-cell contacts. Furthermore, the midbody itself has been implicated in controlling proliferation via EGF and integrin signaling in HeLa cells (Peterman et al., 2019) and in controlling stemness of neural progenitors via the midbody protein prominin-1 (Dubreuil et al., 2007). The exact mechanisms by which intercellular bridges and midbodies affect ES cell fate will be an interesting question for future studies.

It will also be interesting to further explore how the abscission machinery is remodeled as cells exit the ES cell state. Our data suggest a role for the ESCRT-III protein CHMP4B, a key driver in the physical resolution of the bridge (reviewed in Stoten and Carlton [2018]), which is progressively recruited to the bridges after induction of naive pluripotency exit. Our data also suggest

(D) Schematic representation of the laser ablation experiment: 5 h after inducing naive pluripotency exit, a tubulin bridge connecting two cells is ablated using a pulsed laser (red thunderbolt), and REX1-GFP levels in the 2 cells (dark gray and yellow) are monitored.
(E) Representative example of a bridge laser ablation experiment in cells labeled with SIR-tubulin (inverted contrast). Ablation is highlighted with a red thunderbolt and the location of the ablated bridge and destroyed midbody is marked with a red star. One Z plane is shown. Scale bar: 10 μm.
(F) Time-lapse confocal microscopy images of the REX1-GFP (green) GAP43-mCherry (magenta) expressing ES cells pictured in (E) after bridge ablation. Red star: location of the ablated bridge. Time in hours. Ablation took place at 5.30 h after 2i/LIF removal. A single Z plane is shown. Dark gray and yellow stars highlight sister cells initially connected by the bridge; light gray, blue, brown, and orange stars highlight granddaughter cells after one further division. Scale bar: 10 μm.
(G) REX1-GFP mean intensity for the ablated cells pictured in (E and F) as a function of time. 0 h: time of 2i/LIF removal. Lines are fitting curves and the time of REX1-GFP downregulation is determined from the first inflection point (see STAR Methods).
(H) Dot plot showing the time of REX1-GFP downregulation for controls cells (gray) and cells with ablated bridges that keep the midbody after ablation (pink) or not (red, either because the midbody was destroyed or because it stayed associated with the other cell). Mean and standard error of the mean are plotted. n = 30 cells for controls, n = 15 cells for "ablation keeps midbody" and n = 15 cells for "ablation does not keep midbody," from n = 18 divisions. N = 4.

that the faster recruitment of CHMP4B during exit from naive pluripotency results from faster structural changes of the bridge. Indeed, in naive cells, bridge thinning is slower than in cells exiting the ES cell state. Since recruitment of ESCRT-III components, including CHMP4B, is thought to depend on the bridge becoming sufficiently narrow, accelerated bridge thinning in cells exiting naive pluripotency could directly result in faster CHMP4B recruitment. Interestingly, in a recent study we also identified a decrease in plasma membrane tension as a key regulator of naive pluripotency exit (De Belly et al., 2019). High membrane tension has been shown to act as a negative regulator of abscission in HeLa cells by preventing the recruitment of ESCRT-III proteins (Lafaurie-Janvore et al., 2013). It is tempting to speculate that the membrane tension decrease during naive pluripotency exit could contribute to the regulation ESCRT machinery recruitment to the bridge. Importantly, membrane tension regulating naive pluripotency exit directly, via endocytosis (De Belly et al., 2019), and abscission affecting pluripotency exit directly, as shown here, are not mutually exclusive. We speculate that De Belly et al. (2019) and the current study identify two distinct, though possibly partly co-regulated, cellular processes contributing to naive pluripotency exit regulation.

In conclusion, our data uncover how changes in a key cell biology process, the separation of sister cells during abscission, acts as a permissive cue for naive pluripotency exit. These results shed light on how modulating the dynamics of specific cell-cycle processes can contribute to cell fate transitions.

STAR★METHODS

Detailed methods are provided in the online version of this paper and include the following:

- KEY RESOURCES TABLE
- RESOURCE AVAILABILITY
    - ○ Lead Contact
    - ○ Materials Availability
    - ○ Data and Code Availability
- EXPERIMENTAL MODEL AND SUBJECT DETAILS
- METHOD DETAILS
    - ○ Cell Culture, Transfection and Live Imaging
    - ○ Cell Sorting
    - ○ Clonogenicity Analysis
    - ○ REX1-GFP Intensity Measurements in Colonies and Analysis
    - ○ Immunofluorescence
    - ○ Microchannel Experiments
    - ○ Single Cell RNA Sequencing
    - ○ Volume Measurements
    - ○ qPCR
    - ○ Photobleaching Experiments
    - ○ Electron Microscopy
    - ○ Post-mitotic Bridge Ablation
- QUANTIFICATION AND STATISTICAL ANALYSIS

SUPPLEMENTAL INFORMATION

Supplemental Information can be found online at https://doi.org/10.1016/j.devcel.2020.09.001.

ACKNOWLEDGMENTS

We thank the entire Paluch, Baum, and Chalut labs for discussions and feedback throughout the project, the LMCB Light Microscopy Facility, in particular Andrew Vaughan, the LMCB Electron Microscopy Facility, and the UCL Flow Cytometry Platform, in particular, Stephanie Canning, for their invaluable expertise and support. A.C. would like to particularly thank Buzz Baum and Gautam Dey for helpful discussions, Moona Huttunen for technical advice and Siân Culley for help with the graphical abstract.. We thank Jonathan Chubb (MRC-LMCB) and Carla Mulas (SCI, University of Cambridge) for providing technical expertise and advice. We thank Stanley Strawbridge (Austin Smith lab, SCI, University of Cambridge) for the GAP43-mCherry construct and for discussions of unpublished data.

This work was supported by the Medical Research Council UK (MRC Program award MC_UU_12018/5), the European Research Council (starting grant 311637-MorphoCorDiv and consolidator grant 820188-NanoMechShape to E.K.P.), and the Leverhulme Trust (Leverhulme Prize in Biological Sciences to E.K.P.). K.J.C. acknowledges support from the Royal Society (Royal Society Research Fellowship). A.C. acknowledges support from EMBO (ALTF 2015-563), the Wellcome Trust (201334/Z/16/Z), and the Fondation Bettencourt-Schueller (Prix Jeune Chercheur, 2015).

AUTHOR CONTRIBUTIONS

A.C. and E.K.P. designed the research and wrote the manuscript; A.C. performed all the experiments and analyzed the data. C.L. performed the single-cell RNA sequencing analysis. E.H. analyzed the REX1-GFP tracking data. M.A. provided technical support. I.J.W. performed the electron microscopy. K.J.C. and E.K.P. supervised the project. All authors provided inputs on the project and the manuscript.

DECLARATION OF INTERESTS

The authors declare no competing interests.

Received: February 20, 2020
Revised: June 22, 2020
Accepted: August 30, 2020
Published: September 25, 2020

REFERENCES

Adell, M.A.Y., and Teis, D. (2011). Assembly and disassembly of the ESCRT-III membrane scission complex. FEBS Lett. *585*, 3191–3196.

Alié, A., Leclère, L., Jager, M., Dayraud, C., Chang, P., Le Guyader, H., Quéinnec, E., and Manuel, M. (2011). Somatic stem cells express Piwi and vasa genes in an adult ctenophore: ancient association of "germline genes" with stemness. Dev. Biol. *350*, 183–197.

Almonacid, M., Terret, M.É., and Verlhac, M.H. (2014). Actin-based spindle positioning: new insights from female gametes. J. Cell Sci. *127*, 477–483.

Anders, S., Pyl, P.T., and Huber, W. (2015). HTSeq–a Python framework to work with high-throughput sequencing data. Bioinformatics *31*, 166–169.

Bergert, M., Erzberger, A., Desai, R.A., Aspalter, I.M., Oates, A.C., Charras, G., Salbreux, G., and Paluch, E.K. (2015). Force transmission during adhesion-independent migration. Nat. Cell Biol. *17*, 524–529.

Brown, K., Loh, K.M., and Nusse, R. (2017). Live imaging reveals that the first division of differentiating human embryonic stem cells often yields asymmetric fates. Cell Rep. *21*, 301–307.

Cabernard, C., and Doe, C.Q. (2009). Apical/basal spindle orientation is required for neuroblast homeostasis and neuronal differentiation in Drosophila. Dev. Cell *17*, 134–141.

Cadart, C., Monnier, S., Grilli, J., Sáez, P.J., Srivastava, N., Attia, R., Terriac, E., Baum, B., Cosentino-Lagomarsino, M., and Piel, M. (2018). Size control in mammalian cells involves modulation of both growth rate and cell cycle duration. Nat. Commun. *9*, 3275.

Cadart, C., Zlotek-Zlotkiewicz, E., Le Berre, M., Piel, M., and Matthews, H.K. (2014). Exploring the function of cell shape and size during mitosis. Dev. Cell *29*, 159–169.

Cannon, D., Corrigan, A.M., Miermont, A., McDonel, P., and Chubb, J.R. (2015). Multiple cell and population-level interactions with mouse embryonic stem cell heterogeneity. Development *142*, 2840–2849.

Carlton, J.G., and Martin-Serrano, J. (2007). Parallels Between cytokinesis and retroviral budding: A role for the ESCRT machinery. Science *316*, 1908–1912.

Chiaruttini, N., Redondo-Morata, L., Colom, A., Humbert, F., Lenz, M., Scheuring, S., and Roux, A. (2015). Relaxation of loaded ESCRT-III spiral springs drives membrane deformation. Cell *163*, 866–879.

Chugh, P., Clark, A.G., Smith, M.B., Cassani, D.A.D., Dierkes, K., Ragab, A., Roux, P.R., Charras, G., Salbreux, G., and Paluch, E.K. (2017). Actin cortex architecture regulates cell surface tension. Nat. Cell Biol. *19*, 689–697.

Coggins, L.W. (1973). An ultrastructural and radioautographic study of early oogenesis in the toad Xenopus laevis. J. Cell Sci. *12*, 71–93.

Cowan, C.R., and Hyman, A.A. (2004). Asymmetric cell division in C. elegans: cortical polarity and spindle positioning. Annu. Rev. Cell Dev. Biol. *20*, 427–453.

Cunningham, F., Amode, M.R., Barrell, D., Beal, K., Billis, K., Brent, S., Carvalho-Silva, D., Clapham, P., Coates, G., Fitzgerald, S., et al. (2015). Ensembl 2015. Nucleic Acids Res. *43*, D662–D669.

David, C.N. (2012). Interstitial stem cells in Hydra: multipotency and decision-making. Int. J. Dev. Biol. *56*, 489–497.

De Belly, H., Jones, P.H., Paluch, E.K., and Chalut, K.J. (2019). Membrane tension mediated mechanotransduction drives fate choice in embryonic stem cells. bioRxiv https://www.biorxiv.org/content/10.1101/798959v1.

Deerinck, T., Bushong, E., Lev-Ram, V., Shu, X., Tsien, R., and Ellisman, M. (2010). Enhancing serial block-face scanning electron microscopy to enable high resolution 3-D nanohistology of cells and tissues. Microsc. Microanal. *16*, 1138–1139.

Dimitracopoulos, A., Srivastava, P., Chaigne, A., Win, Z., Shlomovitz, R., Lancaster, O.M., Le Berre, M., Piel, M., Franze, K., Salbreux, G., and Baum, B. (2020). Mechanochemical crosstalk produces cell-intrinsic patterning of the cortex to orient the mitotic spindle. Curr. Biol. https://www.sciencedirect.com/science/article/pii/S0960982220309842.

Dubreuil, V., Marzesco, A.M., Corbeil, D., Huttner, W.B., and Wilsch-Bräuninger, M. (2007). Midbody and primary cilium of neural progenitors release extracellular membrane particles enriched in the stem cell marker prominin-1. J. Cell Biol. *176*, 483–495.

Ettinger, A.W., Wilsch-Bräuninger, M., Marzesco, A.M., Bickle, M., Lohmann, A., Maliga, Z., Karbanová, J., Corbeil, D., Hyman, A.A., and Huttner, W.B. (2011). Proliferating versus differentiating stem and cancer cells exhibit distinct midbody-release behaviour. Nat. Commun. *2*, 503.

Gershony, O., Pe'er, T., Noach-Hirsh, M., Elia, N., and Tzur, A. (2014). Cytokinetic abscission is an acute G1 event. Cell Cycle *13*, 3436–3441.

Gonzales, K.A., Liang, H., Lim, Y.S., Chan, Y.S., Yeo, J.C., Tan, C.P., Gao, B., Le, B., Tan, Z.Y., Low, K.Y., et al. (2015). Deterministic restriction on pluripotent state dissolution by cell-cycle pathways. Cell *162*, 564–759.

Guizetti, J., Schermelleh, L., Mäntler, J., Maar, S., Poser, I., Leonhardt, H., Müller-Reichert, T., and Gerlich, D.W. (2011). Cortical constriction during abscission involves helices of ESCRT-III-dependent filaments. Science *331*, 1616–1620.

Habib, S.J., Chen, B.C., Tsai, F.C., Anastassiadis, K., Meyer, T., Betzig, E., and Nusse, R. (2013). A localized Wnt signal orients asymmetric stem cell division in vitro. Science *339*, 1445–1448.

Hu, C.K., Coughlin, M., and Mitchison, T.J. (2012). Midbody assembly and its regulation during cytokinesis. Mol. Biol. Cell *23*, 1024–1034.

Kalkan, T., Olova, N., Roode, M., Mulas, C., Lee, H.J., Nett, I., Marks, H., Walker, R., Stunnenberg, H.G., Lilley, K.S., et al. (2017). Tracking the embryonic stem cell transition from ground state pluripotency. Development *144*, 1221–1234.

Lafaurie-Janvore, J., Maiuri, P., Wang, I., Pinot, M., Manneville, J.B., Betz, T., Balland, M., and Piel, M. (2013). ESCRT-III assembly and cytokinetic abscission are induced by tension release in the intercellular bridge. Science *339*, 1625–1629.

Liu, L., Michowski, W., Inuzuka, H., Shimizu, K., Nihira, N.T., Chick, J.M., Li, N., Geng, Y., Meng, A.Y., Ordureau, A., et al. (2017). G1 cyclins link proliferation, pluripotency and differentiation of embryonic stem cells. Nat. Cell Biol. *19*, 177–188.

Love, M.I., Huber, W., and Anders, S. (2014). Moderated estimation of fold change and dispersion for RNA-seq data with DESeq2. Genome Biol. *15*, 550.

Lukinavičius, G., Reymond, L., D'Este, E., Masharina, A., Göttfert, F., Ta, H., Güther, A., Fournier, M., Rizzo, S., Waldmann, H., et al. (2014). Fluorogenic probes for live-cell imaging of the cytoskeleton. Nat. Methods *11*, 731–733.

Lun, A.T.L., McCarthy, D.J., and Marioni, J.C. (2016). A step-by-step workflow for low-level analysis of single-cell RNA-seq data with bioconductor. F1000Res. *5*, 2122.

Maître, J.L., Turlier, H., Illukkumbura, R., Eismann, B., Niwayama, R., Nédélec, F., and Hiiragi, T. (2016). Asymmetric division of contractile domains couples cell positioning and fate specification. Nature *536*, 344–348.

Martello, G., and Smith, A. (2014). The nature of embryonic stem cells. Annu. Rev. Cell Dev. Biol. *30*, 647–675.

Matias, N.R., Mathieu, J., and Huynh, J.R. (2015). Abscission is regulated by the ESCRT-III protein shrub in Drosophila germline stem cells. PLoS Genet. *11*, e1004653.

Matt, G., and Umen, J. (2016). Volvox: a simple algal model for embryogenesis, morphogenesis and cellular differentiation. Dev. Biol. *419*, 99–113.

Mierzwa, B.E., Chiaruttini, N., Redondo-Morata, L., von Filseck, J.M., König, J., Larios, J., Poser, I., Müller-Reichert, T., Scheuring, S., Roux, A., and Gerlich, D.W. (2017). Dynamic subunit turnover in ESCRT-III assemblies is regulated by Vps4 to mediate membrane remodelling during cytokinesis. Nat. Cell Biol. *19*, 787–798.

Mierzwa, B., and Gerlich, D.W. (2014). Cytokinetic abscission: molecular mechanisms and temporal control. Dev Cell. *31*, 525–538.

Morita, E., Sandrin, V., Chung, H.Y., Morham, S.G., Gygi, S.P., Rodesch, C.K., and Sundquist, W.I. (2007). Human ESCRT and ALIX proteins interact with proteins of the midbody and function in cytokinesis. EMBO J. *26*, 4215–4227.

Mort, R.L., Ford, M.J., Sakaue-Sawano, A., Lindstrom, N.O., Casadio, A., Douglas, A.T., Keighren, M.A., Hohenstein, P., Miyawaki, A., and Jackson, I.J. (2014). Fucci2a: a bicistronic cell cycle reporter that allows Cre mediated tissue specific expression in mice. Cell Cycle *13*, 2681–2696.

Mulas, C., Kalkan, T., and Smith, A. (2017). NODAL secures pluripotency upon embryonic stem cell progression from the ground state. Stem Cell Reports *9*, 77–91.

Mulas, C., Kalkan, T., von Meyenn, F., Leitch, H.G., Nichols, J., and Smith, A. (2019). Defined conditions for propagation and manipulation of mouse embryonic stem cells. Development *146*, dev173146.

Ou, G., Stuurman, N., D'Ambrosio, M., and Vale, R.D. (2010). Polarized myosin produces unequal-size daughters during asymmetric cell division. Science *330*, 677–680.

Pauklin, S., and Vallier, L. (2013). The cell-cycle state of stem cells determines cell fate propensity. Cell *155*, 135–147.

Pepling, M.E., de Cuevas, M., and Spradling, A.C. (1999). Germline cysts: a conserved phase of germ cell development? Trends Cell Biol. *9*, 257–262.

Pepling, M.E., and Spradling, A.C. (1998). Female mouse germ cells form synchronously dividing cysts. Development *125*, 3323–3328.

Peterman, E., Gibieža, P., Schafer, J., Skeberdis, V.A., Kaupinis, A., Valius, M., Heiligenstein, X., Hurbain, I., Raposo, G., and Prekeris, R. (2019). The post-abscission midbody is an intracellular signaling organelle that regulates cell proliferation. Nat. Commun. *10*, 3181.

Picelli, S., Faridani, O.R., Björklund, A.K., Winberg, G., Sagasser, S., and Sandberg, R. (2014). Full-length RNA-seq from single cells using Smart-seq2. Nat. Protoc. *9*, 171–181.

Saini, D., and Yamanaka, Y. (2018). Cell polarity-dependent regulation of cell allocation and the first lineage specification in the preimplantation mouse embryo. Curr. Top. Dev. Biol. *128*, 11–35.

Schneider, C.A., Rasband, W.S., and Eliceiri, K.W. (2012). NIH image to imageJ: 25 years of image analysis. Nat Methods. *9*, 671–675.

Schindelin, J., Arganda-Carreras, I., Frise, E., Kaynig, V., Longair, M., Pietzsch, T., Preibisch, S., Rueden, C., Saalfeld, S., Schmid, B., et al. (2012). Fiji: an open-source platform for biological-image analysis. Nat. Methods *9*, 676–682.

Scialdone, A., Natarajan, K.N., Saraiva, L.R., Proserpio, V., Teichmann, S.A., Stegle, O., Marioni, J.C., and Buettner, F. (2015). Computational assignment of cell-cycle stage from single-cell transcriptome data. Methods *85*, 54–61.

Simons, B.D., and Clevers, H. (2011). Strategies for homeostatic stem cell self-renewal in adult tissues. Cell *145*, 851–862.

Smith, M.B., Chaigne, A., and Paluch, E.K. (2017). An active contour ImageJ plugin to monitor daughter cell size in 3D during cytokinesis. Methods Cell Biol. *137*, 323–340.

Soncin, F., Mohamet, L., Eckardt, D., Ritson, S., Eastham, A.M., Bobola, N., Russell, A., Davies, S., Kemler, R., Merry, C.L.R., and Ward, C.M. (2009). Abrogation of E-cadherin-mediated cell-cell contact in mouse embryonic stem cells results in reversible LIF-independent self-renewal. Stem Cells *27*, 2069–2080.

Soufi, A., and Dalton, S. (2016). Cycling through developmental decisions: how cell cycle dynamics control pluripotency, differentiation and reprogramming. Development *143*, 4301–4311.

Stoten, C.L., and Carlton, J.G. (2018). ESCRT-dependent control of membrane remodelling during cell division. Semin. Cell Dev. Biol. *74*, 50–65.

Strawbridge, S.E., Blanchard, G.B., Smith, A., Kugler, H., and Martello, G. (2020). Embryonic stem cells commit to differentiation by symmetric divisions following a variable lag period. bioRxiv. https://doi.org/10.1101/2020.06.17.157578.

Waisman, A., Vazquez Echegaray, C., Solari, C., Cosentino, M.S., Martyn, I., Deglincerti, A., Ozair, M.Z., Ruzo, A., Barañao, L., Miriuka, S., et al. (2017). Inhibition of cell division and DNA replication impair mouse-naïve pluripotency exit. J. Mol. Biol. *429*, 2802–2815.

Williams, S.E., and Fuchs, E. (2013). Oriented divisions, fate decisions. Curr. Opin. Cell Biol. *25*, 749–758.

Wu, T.D., and Nacu, S. (2010). Fast and SNP-tolerant detection of complex variants and splicing in short reads. Bioinformatics *26*, 873–881.

Yang, P., Humphrey, S.J., Cinghu, S., Pathania, R., Oldfield, A.J., Kumar, D., Perera, D., Yang, J.Y.H., James, D.E., Mann, M., and Jothi, R. (2019). Multi-omic profiling reveals dynamics of the phased progression of pluripotency. Cell Syst. *8*, 427–445.e10.

Zenker, J., White, M.D., Templin, R.M., Parton, R.G., Thorn-Seshold, O., Bissiere, S., and Plachta, N. (2017). A microtubule-organizing center directing intracellular transport in the early mouse embryo. Science *357*, 925–928.

CellPress
OPEN ACCESS

STAR★METHODS

KEY RESOURCES TABLE

REAGENT or RESOURCE	SOURCE	IDENTIFIER
Antibodies		
a-Tubulin	Thermo Fischer	Cat#62204; RRID: AB_1965960
a-Tubulin	Thermo Fischer	Cat#MA180017; RRID: AB_2210201
CRIK	Insight Biotechnology	Cat#611376; RRID: AB_398899
Alexa Fluor® 647-AffiniPure Donkey Anti-Rat IgG	Stratech Scientific	Cat#712-605-153-JIR; RRID: AB_2340694
Donkey anti-Mouse IgG (H+L) Highly Cross-Adsorbed Secondary Antibody, Alexa Fluor 488	Thermo Fisher Scientific	Cat#A-21202; RRID: AB_141607
Chemicals, Peptides, and Recombinant Proteins		
SIR-Tubulin	Tebu-bio	Cat#SC002
B27	Life technologies	Cat#12587010
CHIRON	Cambridge Bioscience	Cat#CAY13122
PD 0325901	Sigma-Aldrich	Cat#PZ0162
LIF	Merck Millipore	Cat# ESG1107
Insulin zinc	Sigma-Aldrich	Cat#I9278
Apotransferrin	Sigma-Aldrich	Cat# T1147
Putrescine	Sigma-Aldrich	Cat#P5780
Sodium Selenite	Sigma-Aldrich	Cat#S5261
Progesterone	Sigma-Aldrich	Cat#P8783
CellMask™ deep red	Thermofisher Scientific	Cat# C10046
Laminin	Sigma-Aldrich	Cat#11243217001
Lipofectamin™ RNAimax	Thermofischer Scientific	Cat# 13778075
Accutase	Sigma-Aldrich	Cat#A6964
DMEM/F-12, 1:1 mixture	Sigma-Aldrich,	Cat#D6421-6
Neurobasal medium	Life technologies	Cat#21103-049
RO-3306	Sigma Aldrich	Cat#SML0569
5MG-Lipofectamine® 2000 Transfection Reagent	Life technologies	Cat#11668-027
Critical Commercial Assays		
High-Capacity cDNA Reverse Transcription Kit	Thermofischer Scientific	Cat#4368814
SsoAdvanced™ Universal SYBR® Green Supermix	BioRad	Cat#172-5271
Deposited Data		
Single cell RNA seq: GEO accession number GEO: GSE14181	https://www.ncbi.nlm.nih.gov/geo/query/acc.cgi?acc=GSE141811	N
Experimental Models: Cell Lines		
Mouse embryonic stem cells: E14	Chalut lab (Cambridge Stem cell Institute, Cambridge, UK)	N/A
Mouse embryonic stem cells: E14 H2B-RFP	Chubb lab (MRC LMCB, University College London, UK)	Cannon et al., 2015
Mouse embryonic stem cells: E14 REX1-GFP, GAP43-mCherry	Smith lab (Cambridge Stem Cell Institute, Cambridge, UK)	Strawbridge et al., 2020
Mouse embryonic stem cells: E14 Fucci2a	Jackson lab (the University of Edinburgh, UK)	Mort et al., 2014

(*Continued on next page*)

Continued

REAGENT or RESOURCE	SOURCE	IDENTIFIER
HeLa cells: H2B-mCherry Tubulin-GFP	Baum lab (MRC LMCB, University College London, UK)	Dimitracopoulos et al., 2020
E14 REX1-GFP	Chalut lab (Cambridge Stem Cell Institute, Cambridge, UK)	Kalkan et al., 2017
S-HeLa cells	Paluch lab (MRC LMCB, University College London, UK)	Chugh et al., 2017
Oligonucleotides		
SMARTpool:ON-TARGETplus Pdcd6ip	Dharmacon	Cat#L-062173-01-0005
SMARTpool:ON-TARGETplus Cep55	Horizon Discovery	Cat# L-044799-01-0005
ON-TARGETplus Non-targeting Pool	Dharmacon	Cat#D-001810-10-05
Primers for Rex1, Klf2, Nanog, Klf4, Fgf5, Otx2, ESRRB, Alix, Cep55, ActB : see Table S2	Integrated DNA technologies	NA
Software and Algorithms		
DeformingMesh3D	(Smith et al., 2017)	https://github.com/PaluchLabUCL/DeformingMesh3D-plugin
ImageJ/Fiji	(Schneider et al., 2012; Schindelin et al., 2012)	https://imagej.nih.gov/ij/
Mouse genome build GRCm38/mm10, GSNAP version 2015-09-29	(Wu and Nacu, 2010)	https://www.ncbi.nlm.nih.gov/assembly/GCF_000001635.20/
Ensembl release 81	(Cunningham et al., 2015)	https://www.ebi.ac.uk/about/news/service-news/ensemblversion-81-release
HTSeq	(Anders et al., 2015)	https://htseq.readth edocs.io/en/master/
scran package in R	(Lun et al., 2016)	https://www.rdocumentation.org/packages/scran/versions/1.0.3
DESeq2 package in R	(Love et al., 2014)	https://bioconductor.org/packages/release/bioc/html/DESeq2.html
Prism 7	Graphpad software, Inc	N/A

RESOURCE AVAILABILITY

Lead Contact

Further information and requests for resources and reagents should be directed to and will be fulfilled by the Lead Contact, Ewa K Paluch (ekp25@cam.ac.uk).

Materials Availability

This study did not generate new unique reagents.

Data and Code Availability

The accession number for the single-cell RNA sequencing data reported in this paper is GEO: GSE141811 (accessible through https://www.ncbi.nlm.nih.gov/geo/query/acc.cgi?acc=GSE141811)

EXPERIMENTAL MODEL AND SUBJECT DETAILS

In this study, the cells used were: E14 wild type ES cells, E14 ES cells stably expressing H2B-RFP, a kind gift from Jonathan Chubb (Cannon et al., 2015), E14 ES cells stably expressing REX1-GFP and GAP43-mCherry (a kind gift from Carla Mulas and Stanley Strawbridge, Austin Smith lab, Stem Cell Institute, University of Cambridge (Strawbridge et al., 2020)), E14 cells stably expressing REX1-GFP (Kalkan et al., 2017), E14 cells expressing the Fucci2a system (Mort et al., 2014) (a kind gift from Ian James Jackson, the University of Edinburgh), HeLa cells expressing H2B-mCherry and Tubulin-GFP (Dimitracopoulos et al., 2020) (a kind gift from Buzz Baum, MRC LMCB, University College London), and suspension HeLa cells (Chugh et al., 2017).

METHOD DETAILS

Cell Culture, Transfection and Live Imaging

HeLa cells were cultured in Dulbecco's Modified Eagles Medium (DMEM GlutaMAX; Sigma #D5796) supplemented with 10% FBS and 50 U/ml penicillin and 50 μg/ml streptomycin at 37 °C under 5% CO_2.

Mouse ES cells were routinely cultured as described in (Mulas et al., 2019) (and see below) on 0.1% gelatin in PBS (unless otherwise stated) in N2B27+2i+LIF + penicillin and streptomycin, at a controlled density (1.5-3.0 10^4 cells/cm^2) in Falcon flasks and passaged every other day using Accutase (Sigma-Aldrich, #A6964). They were kept in 37°C incubators with 7% CO_2. Cells were regularly tested for mycoplasma.

The culture medium was made in house, using DMEM/F-12, 1:1 mixture (Sigma-Aldrich, #D6421-6), Neurobasal medium (Life technologies #21103-049), 2.2 mM L-Glutamin, home-made N2 (see below), 1:50 B27 (Life technologies #12587010), 3 μM Chiron (Cambridge Bioscience #CAY13122), 1 μM PD 0325901 (Sigma-Aldrich #PZ0162), 10 ng.mL^{-1} LIF (Merck Millipore # ESG1107), 50 mM β-Mercapto-ethanol, 12.5 ng.mL^{-1} Insulin zinc (Sigma-Aldrich #I9278). The 200 X home-made N2 was made using 0.791 mg.mL^{-1} Apotransferrin (Sigma-Aldrich #T1147), 1.688 mg.mL^{-1} Putrescine (Sigma-Aldrich #P5780), 3 μM Sodium Selenite (Sigma-Aldrich #S5261), 2.08 μg.mL^{-1} Progesterone (Sigma-Aldrich #P8783), 8.8% BSA. Exit from naïve pluripotency was triggered by passaging the cells and seeding them in N2B27. When indicated RO-3306 (Sigma-Aldrich #CatSML0569) was added at a final concentration of 6 μM.

For colony imaging, the cells were typically plated on 35 mm IbidI dishes (IBI Scientific, #81156) coated with gelatin (unless otherwise stated) the day before the experiment, and imaged on a Perkin Elmer Ultraview Vox spinning disc (Nikon Ti attached to a Yokogawa CSU-X1 spinning disc scan head) using a C9100-13 Hamamatsu EMCCD Camera. Samples were imaged using a 60X water objective (CFI Plan Apochromat with Zeiss Immersol W oil, Numerical Aperture 1.2). Typically, the samples were imaged acquiring a Z-stack with $\Delta Z = 2$ μm.

siRNA treatment was performed using 2.5 μL Lipofectamin™ RNAimax (Thermofischer Scientific, # 13778075) and 1 μL siRNA (20 μmol.L^{-1} for a final concentration of 20 nmol.L^{-1}) each mixed in 250 μL OptiMEM for 5 min, then mixed together and incubated at room temperature for 20 min. 300,000 cells were then resuspended, and plated in a 12-well plate in 500 μL media total + siRNA mix. The cells were incubated with siRNA for 24h before experiments and qPCR. The RNA used were SMARTpool:ON-TARGETplus Pdcd6ip (Dharmacon #L-062173-01-0005) for ALIX depletion, SMARTpool:ON-TARGETplus Cep55 (Horizon Discovery # L-044799-01-0005) for CEP55 depletion, and ON-TARGETplus Non-targeting Pool (Dharmacon #D-001810-10-05) as a scrambled control. When indicated Dendra2-H3.3-N-14 (Addgene #57725) was transfected with a similar protocole using Lipofectamine® 2000 Transfection Reagent (Life technologies #11668-027).

For live imaging of the spindle and post-mitotic bridges, tubulin was labeled using SIR-Tubulin (Tebu-bio #SC002, diluted in media to 20 nM and incubated for 6h then rinsed). These conditions were chosen because they allowed an optimal tubulin staining while not stabilizing the microtubules (as assessed by a normal duration of cell division).

When specified, Ibidi dishes (IBI Scientific, #81156) were incubated overnight with 10 μg.mL^{-1} Laminin (Sigma, #11243217001) at 37°C.

Cell Sorting

Cells were sorted according to the fluorescence levels or forward scatter and side scatter to sort the cells by size (this recapitulates cell-cycle sorting (see Figure S2A)) using FACSAria III Cell Sorter at the UCL flow cytometry core facility at UCL Great Ormond Street Institute of Child Health.

Clonogenicity Analysis

To test for speed and efficiency of exit from naïve pluripotency, replating assays were performed. After various treatments such as sorting or siRNA treatments, the cells were plated at low density (30,000 cells per well of a 24-well plate) onto plates coated with 0.1% gelatin in N2B27 for 26 hours. Then the cells were resuspended, counted, and replated at low density (200 cells per well of a 12-well plate) on 0.1% gelatin in N2B27+2i+LIF. After 5 days, the number of colonies was manually counted.

REX1-GFP Intensity Measurements in Colonies and Analysis

ES cells stably expressing REX1-GFP and GAP43-mCherry were plated in N2B27 on 0.1% gelatin-coated IbidI dishes and 4 hours after plating, Z-stacks with $\Delta Z = 2$ μm were acquired. REX1-GFP mean intensity was manually measured in the cytoplasm at the mid-plane of the cell using a rectangular region of interest for each cell at each time point using Fiji (Schindelin et al., 2012).

To determine whether the correlation between division and naïve pluripotency exit (Figure S1I) could be due to chance, we used a non-parametric bootstrapping method. We first fitted the REX1 intensity curves to a sigmoidal decay function and extracted the time of naïve pluripotency exit τ. We discarded the time courses for which this time could not be accurately determined (i.e. those where fitting the REX1 time course gave an error of fit for τ that was on the order of the value of τ itself). We then calculated the coefficient of determination R^2 of the linear regression between the time of naïve pluripotency exit and the time of cell division (in cases where there were two events of division, the one closest to the time of naïve pluripotency exit was picked). This gave R^2=0.73 for the points that passed the criterion. We then bootstrapped the dataset by randomly assigning the time of naïve pluripotency exit of a cell i to the time(s) of cell division of a randomly selected cell j (the procedure was done with replacement) and calculated the coefficient of

determination R^2 for the randomized dataset. We performed this procedure 1000 times to build a distribution of probability of correlation values. Importantly, we found that the observed correlation occurred just less than 5% of the time, underlying its statistical significance (Figure S1I).

To determine the extent to which the dynamics of REX1 downregulation in daughter cells were correlated (Figures 3E and 3F), we first separated cells exiting naïve pluripotency at the first division (where correlation between daughters was analyzed prior to them dividing again), and cells exiting naïve pluripotency at the second division (where correlation between grand-daughters was considered). To avoid artefacts due to differences in REX1 expression levels between cells, we first normalized all REX1 curves so that their first time point is of intensity 1. We first calculated for each time point, the average decrease of REX1 intensity across all cells, as well as the standard deviation around it. This gave us a population-average of the variance ("global variance") that would be observed if cells had no correlation from being sisters. We then computed the variance at each time point between the REX1 curves of two sisters ("local variance"). In both "global" and "local" case, we then averaged across time the variances, and compared the results. We only found two cases in which sister-sister variance was larger than the population average, which were the only two cases in which two sisters exited naïve pluripotency at different times. Importantly, looking at the full dataset, we found that the average variance between sisters was typically 2-3-fold smaller than the global population variance (Figure 3F), showing that sisters display significant correlation in REX1 downregulation dynamics.

Immunofluorescence

Cells were fixed in IbidI dishes (IBI Scientific, #81156) in 4% formaldehyde in PHEM buffer with 0.125% Triton. Primary antibodies against α-Tubulin (Thermo Fischer #62204 or Thermo Fischer Scientific #MA180017), CRIK (Insight Biotechnology #611376) were incubated 1:200 in PBS with 5% non-fat dry Milk for 2h at room temperature, and the secondary antibody was incubated 1:500 for 1h at room temperature. Secondary antibodies were: Alexa Fluor® 647-AffiniPure Donkey Anti-Rat IgG (Stratech Scientific, #712-605-153-JIR), Donkey anti-Mouse IgG (H+L) Highly Cross-Adsorbed Secondary Antibody, Alexa Fluor 488 (Thermo Fisher Scientific, #A-21202). The cells were mounted using ProLong® Gold Antifade Mountant with 1:10,000 DAPI (Thermofisher Scientific, #P36941) and imaged using a 63X HCX PL APO (Numerical Aperture 0.6 - 1.4) on a confocal microscope (Leica DMI6000 Microscope).

Microchannel Experiments

PDMS microchannels were fabricated as previously described (Bergert et al., 2015). Briefly, PDMS was polymerized over wafers for 8 μm * 5 μm or 10 μm * 10 μm channels and over 35 -mm coverslips and pre-baked at 60°C. Holes were punched on top of the channels then all the parts were coated with PDMS and attached to a dish and baked overnight at 60°C. The channels were filled with media using syringes and left to equilibrate for 1h at 37 °C and all bubbles were removed by softly pushing the gel down before the cells were injected using syringes.

For cells in microchannels, REX1-GFP mean intensity was manually measured in the midplane of the cell every hour using a rectangular selection in the cell cytoplasm for each cell using Fiji (Schindelin et al., 2012).

Single Cell RNA Sequencing

RNA Sequencing and Analysis

Library preparation was done by the Stem Cell Institute Genomics Facility using SmartSeq2 method and Nextera XT kits (Illumina) (Picelli et al., 2014). Paired-end sequencing was performed on Illumina HiSeq4000 yielding 380 Million reads per lane.

RNA Data Processing and Transcriptome Analysis

Mouse genome build GRCm38/mm10 was used to align reads using GSNAP version 2015-09-29 (Wu and Nacu, 2010). Genes were annotated using Ensembl release 81 (Cunningham et al., 2015) and read counts were quantified using HTSeq (Anders et al., 2015). Quality control and downstream analyses were performed using scran package in R (Lun et al., 2016). Expression was computed using DESeq2 package in R (Love et al., 2014) with p-adjusted<0.05. Log-transformed normalized counts were used for subsequent heatmaps and expression plots. The cyclone function of the scran package was used to assign a cell-cycle phase to individual cells (Scialdone et al., 2015). In Figure S2C, the expression levels of the top 50 highly expressed genes involved in stem cell population maintenance genes (GO: 0019827) were assessed.

Clustering of Cells Based on Expression of Two Pluripotency Genes Tfcp2l1 *and* Tbx3

The two genes *Tfcp2l1* and *Tbx3* were selected because they are among the first genes to be downregulated during exit from naïve pluripotency (Kalkan et al., 2017), and they had the highest variation among all naïve pluripotency genes when compared to naïve cells. Cells were assigned to one of 4 clusters using the k-mean clustering method, which minimizes the sum of squares of distance of each point to its cluster center. The clusters were then identified as high or low expression of each gene. Clustering was computed on normalized expression using DESeq2 (Love et al., 2014) on both naïve cells and cells exiting naïve pluripotency together.

Volume Measurements

Cell volumes were measured from Z-stacks using the 3D mesh plugin we previously published (Smith et al., 2017), https://github.com/PaluchLabUCL/DeformingMesh3D-plugin. The far-red membrane dye CellMask™ (Thermofisher Scientific, # C10046) was used for cell segmentation. The parameters used for segmentation were determined as the best by visual analysis. The parameters chosen were: gamma: 1000; alpha: 5; pressure: 0; normalize: 5; image weight: 1.0E-4; divisions: 3; curve weight: 0; beta: 0. The mesh

deformation was made according to the perpendicular maximal gradient of the signal. The segmentation was stopped when the volume seemed resolved by visual assessment.

qPCR

RNA extraction was performed using the RNA Easy Qiagen kit according to the manufacturer's instructions. The reverse transcription was performed using the High-Capacity cDNA Reverse Transcription Kit (Thermofischer Scientific #4368814). qPCR was performed using SsoAdvanced™ Universal SYBR® Green Supermix (BioRad, #172-5271), loading 2.3 μg per lane. Primers were bought from Integrated DNA technologies.

Photobleaching Experiments

Cells were transfected with EGFP the day before the experiment and plated on laminin. Photobleaching was performed using an Olympus FluoView FV1200 Confocal Laser Scanning Microscope with 70% 405 nm laser on an ROI comprising most of the cytoplasm of the cell using the laser light stimulation (SIM) scanner with a 60X objective (UPLSAPO60XS, Numerical Aperture 1.3). Images were acquired at full speed (every 1.1s). The mean GFP intensity in a ROI of fixed size was measured on the images. To quantify the amplitude of fluorescence loss following photobleaching, we measured the minimum intensity reached in a sister cell still connected to the photobleached cell by a bridge, or in an unconnected neighbor (Figure 4D). Fluorescence levels were normalized to the initial fluorescence levels, to take into account cell-to-cell variability in GFP expression.

Electron Microscopy

Cells were cultured on gridded coverslip–bottom dishes (MatTek) coated with laminin to facilitate correlation between light and electron microscopy. Bridges were identified by phase contrast in light microscopy. Samples were then prepared for electron microscopy following a protocol adapted from (Deerinck et al., 2010). Briefly, samples were fixed in 2 % PFA/2.5 % Glutaraldehyde solution (EM grade, TAAB) for 30 min at room temperature. Samples were washed in 0.1 M sodium cacodylate buffer and post-fixed in 1% OsO4 for 1h at 4°C. Samples were then stained by application of 1% thiocarbohydrazide for 20 min at room temperature, 2% OsO4 for 30 min at room temperature, 1% uranyl acetate overnight at 4°C and lead aspartate for 30 min at 60°C, with intermediate washing in dH2O. This was followed by dehydration of the samples by graded ethanol incubations in 70%, 90% and 100% ethanol and embedding in epon resin (TAAB). Coverslips removed from the dishes were inverted onto prepolymerized epon stubs and polymerized by baking at 60°C overnight. Coverslips were removed from the polymerized resin by plunging into liquid nitrogen, and the cells of interest were found on the block surface by using the grid marks transferred from the coverslip, and the light microscopy images. Serial 70 nm thin sections were cut with a 45° diamond knife (DiatomeDiATOME) using an ultramicrotome (UC7; Leica). Ribbons of sequential sections were collected on 1 × 2 mm Formvar-coated slot grids, and imaged using a transmission electron microscope (Tecnai G2 Spirit; FEI) and a charge-coupled device camera (SIS Morada; Olympus).

To measure the width of the bridge and the size of the dense midzone of the bridge, we selected the mid-section of the bridge out of the 3D sectioning.

Post-mitotic Bridge Ablation

Cells were plated on laminin the day before the experiment in medium supplemented with 20 nM SIR-Tubulin. Exit from naïve pluripotency was triggered 5h before ablation by changing the medium for N2B27 supplemented with SIR-Tubulin. Ablation was performed using a LSM880 Multiphoton microscope with a Plan Apochromat 40X oil objective (Numerical Aperture 1.3) with the 760 nm pulsed Chameleon Vision II TiSa laser at 50% laser power. The position of the ablation spot with respect to the midbody and the localization of the midbody after ablation were visually assessed using the SIR-Tubulin and transmitted light channels.

QUANTIFICATION AND STATISTICAL ANALYSIS

Prism 7 (Graphpad software, Inc) was used for all statistical analysis. The D'Agostino & Pearson test was used to test for the normal distribution of data. To compare means, a Student t-test, a Student t-test with Welch correction or a Mann-Whitney test were performed if the data were normally distributed with similar standard deviations, normally distributed but with different standard deviations or not normally distributed, respectively. For contingency data, χ^2 tests were performed. For bridges and midbody counting, the data were blinded. In figure legends, N indicates the number of independent experiments, and n the number of points (not stated for dot plots). Pooled independent experiments are presented in dot plots; before pooling experiments, we compared the means of the different replicates using appropriate tests (as listed above).

Developmental Cell, Volume *55*

Supplemental Information

Abscission Couples Cell Division to Embryonic Stem Cell Fate

Agathe Chaigne, Céline Labouesse, Ian J. White, Meghan Agnew, Edouard Hannezo, Kevin J. Chalut, and Ewa K. Paluch

Supplementary Information for

Abscission Couples Cell Division to Embryonic Stem Cell Fate

Agathe Chaigne[1,*], Céline Labouesse[2], Ian J. White[1], Meghan Agnew[1], Edouard Hannezo[3], Kevin J. Chalut[2], Ewa K. Paluch[1,2,4,*,#]

[1] MRC Laboratory for Molecular Cell Biology, University College London, London WC1E 6BT, UK.

[2] Wellcome/MRC Cambridge Stem Cell Institute, University of Cambridge, Cambridge CB2 0AW, UK.

[3] Institute of Science and Technology Austria, 3400 Klosterneuburg, Austria.

[4] Department of Physiology, Development and Neuroscience, University of Cambridge, Cambridge CB2 3DY, UK.

* Correspondence: A.C.: a.chaigne@ucl.ac.uk; E.K.P.: ekp25@cam.ac.uk

[#] Lead author

Supplementary Figure Legends

Figure S1. ES cells exit naïve pluripotency after mitosis (related to Figure 1)

A) Representative confocal microscopy images of ES cells expressing REX1-GFP (green) and GAP43-mCherry (magenta), 5h or 40h after being placed in N2B27 supplemented with 6 μM of RO-3306 or equivalent DMSO volume. Scale bars: 10 μm. B) Dot plot showing REX1-GFP intensity 40h after removal of 2i/LIF for cells treated with 6 μM RO-3306 (purple) or DMSO (control, grey). Mean and standard deviation are shown. N=2. C) Dot plot showing proliferation rate over 24h of ES cells treated with DMSO (grey) or 6 μM RO-3306 (purple). Mean and standard deviation are shown. N=5. D) Representative confocal images showing cell areas 40h after 2i/LIF removal and treatment with DMSO (top) or 6 μM RO-3306 (bottom). GAP43-mCherry (magenta) is shown as a membrane marker. One Z-plane is shown around the middle plane of the cells. E) Dot plot showing cell area 40h after removal of 2i/LIF in presence of DMSO (grey) or 6 μM RO-3306 (purple). Mean and standard deviation are shown. N=2. F) Plot of REX1-GFP mean intensity in cells expressing REX1-GFP and GAP43-mCherry during exit from naïve pluripotency, as a function of time. 0h: time of 2i/LIF removal. Mean and standard error of the mean are plotted. N=3, n= 33. G) Bar graphs showing the expression of key pluripotency genes (left) or genes associated with naïve pluripotency exit (right), as assessed by qPCR, during exit from naïve pluripotency. Values are normalized to RNA levels at 0h. The mean and standard error of the mean are shown. N=3. H) Plot of REX1-GFP mean intensity in naïve ES cells expressing REX1-GFP and GAP43-mCherry, as a function of time before and after cell division. The mean and standard error of the mean are plotted. N=2, n= 11 mother cells and 22 daughter cells. I) Left: schematic outlining the analysis of the robustness of the

correlation between time of naïve pluripotency exit and time of division. Right: cumulative probability of correlation coefficients R^2 (see Methods for details) arising at random (purple), plotted together with the experimental correlation (green). All scale bars: 10 µm.

Figure S2. Inducing naïve pluripotency exit early in the cell cycle leads to faster loss of naïve pluripotency (related to Figure 2)

A) Bar graph showing the percentage of cells in each phase of the cell cycle assessed by comparing FUCCI2a fluorescence in cells sorted by cell size; the “small” cell population is compared to the ungated population (G1: red; G1/S: yellow; S/G2/M: green; black: exit of mitosis). Mean and standard error of the mean are plotted. N=2. B) Bar graph showing the percentage of cells in each phase of the cell cycle, as determined by single cell RNA sequencing analysis, 6h after triggering naïve pluripotency exit in cell populations sorted by size (the “early cell cycle” population corresponds to the cells sorted as small, see (A)). G1: red; S: light green; G2/M: green. C) Heat-map showing the Log2 counts of the levels of expression of the main pluripotency genes for the two cell populations obtained by sorting cells by size, and placed in N2B27 for 6h. Early cell cycle (small cells): pink; ungated: blue. D) Bar graph showing the percentage of cells displaying high or low expression levels of *Tfcp2l1* and *Tbx3*, used to assess exit from naïve pluripotency (see Methods). Early cell cycle: pink; ungated: blue.

Figure S3. Confinement induces strongly asymmetric divisions with no effect on exit from naïve pluripotency dynamics (related to Figure 3)

A) Time-lapse spinning-disk confocal microscopy images of a naïve ES cell expressing H2B-RFP (red) and labeled with CellMask^{TM} deep red (cyan) dividing in a 10*10 µm channel (top) or an 8*5 µm channel (bottom). Time in min; 0 min: anaphase. One Z-plane is shown. Scale bar: 10 µm. B) Dot plot representing the sister cell asymmetry ratio (ratio of cell lengths in the channels) of H2B-RFP ES cells dividing in 8*5 µm, 6*8 µm or 10*10 µm channels. Mean and standard deviation are plotted. N=2 for each condition. C) Graph showing an example of REX1 intensity time course for two daughter cells resulting from an asymmetric division in a 8*5 µm microchannel. 0h: time of division.

Figure S4. Additional experiments investigating abscission dynamics during naïve pluripotency exit and the effects of abscission on exiting the ES cell state (Related to Figure 5)

A) Dot plot showing the fraction of cells associated with a midbody remnant (number of midbody remnants divided by number of cells in a given analysis frame) detected in colonies of naïve H2B-RFP ES cells and during exit from naïve pluripotency. Midbody remnants are defined as CRIK spots not attached to a bridge. Mean and standard error of the mean are shown. N=2. B) Dot plot showing the fraction of cells with bridges (number of bridges divided by number of cells in a given analysis frame) as a function of cell density (quantified as the number of cells per frame of fixed dimension) for cells plated on laminin (growing in 2D colonies) at different times of exit from naïve pluripotency. p-value of Pearson correlation <0.0001. C) Box plot showing RNA levels for H2B-RFP ES cells treated with Scrambled siRNA (*SCR*, black) or siRNA against *Alix* (orange) for 24h in 2i/LIF (Naïve, left) or for 24h in 2i/LIF followed by 6h in N2B27 (Exit, right). Values are normalized to *SCR* controls. Mean and

standard error of the mean are shown. N=5 for 2i/LIF and N=2 for 6h after 2i/LIF removal. D) Box plot showing RNA levels for H2B-RFP ES cells treated with Scrambled siRNA (*SCR*, black) or siRNA against *Alix* (orange) for 24h in 2i/LIF followed by 24h in N2B27. Values are normalized to *SCR* controls. Mean and standard error of the mean are shown. N=5. E) Dot plot showing the proliferation (number of cells recovered divided by number of cells plated) after 24h of culture in 2i/LIF for ES cells treated with Scrambled siRNA (*SCR*, black) or siRNA against *Alix* (orange). Mean and standard deviation are plotted. N=7.

Figure S5. Additional experiments showing that interfering with abscission impairs exit form naïve pluripotency (related to Figure 7)

A) Dot plot showing the fraction of cells with bridges (number of bridges divided by number of cells in a given analysis frame) in naïve H2B-RFP ES cell colonies maintained in Serum/LIF or allowed to exit naïve pluripotency for 24 or 48h. Mean and standard error of the mean are shown. N=2. B) Dot plot showing the fraction of cells associated with a midbody remnant (number of midbody remnants divided by number of cells in a given analysis frame) in naïve H2B-RFP ES cell colonies maintained in Serum/LIF or allowed to exit naïve pluripotency for 24 or 48h. Midbody remnants are defined as CRIK spots not attached to a bridge. Mean and standard error of the mean are shown. N=2. C) Box plot showing RNA levels for H2B-RFP ES cells treated with Scrambled siRNA (*SCR*, black) or siRNA against *Alix* (orange) for 24h in Serum/LIF, then cultured in N2B27 for 24h. Values are normalized to *SCR* controls. Mean and standard error of the mean are shown. N=2. D) Schematic showing the relationship between CEP55, ALIX (and ESCRT-I components, such as TSG101), and ESCRT-III. E) Box plot showing RNA levels in naïve cells treated with Scrambled siRNA (*SCR*,

black) or siRNA against *Cep55* (orange) for 24h. *Klf2* levels are shown as a negative control. Values are normalized to *SCR* controls. Mean and standard error of the mean are shown. N=2. F) Dot plot showing the fraction of cells with bridges (number of bridges divided by number of cells in a given analysis frame) for ES cells treated with siRNA *Scrambled* (*SCR*, grey) or *Cep55* (orange), maintained in 2i/LIF or allowed to exit naïve pluripotency for 24 or 48h. Mean and standard error of the mean are shown. N=2. G) Dot plot showing the fraction of cells associated with a midbody remnant (number of midbody remnants divided by number of cells in a given analysis frame) for ES cells treated with siRNA *Scrambled* (*SCR*, grey) or *Cep55* (orange), maintained in 2i/LIF or allowed to exit naïve pluripotency for 24 or 48h. Midbody remnants are defined as CRIK spots not attached to a bridge. Mean and standard error of the mean are shown. N=2. H) Dot plot representing the number of colonies surviving in a clonogenicity assay (see Figure 2A) for ES cells treated with siRNA *Scrambled* (*SCR*, grey) or *Cep55* (orange) for 24h in 2i/LIF, then placed in differentiation-promoting medium for 24h. Mean and standard error of the mean are shown. N=3.

Figure S6. Triggering premature abscission and midbody release accelerates exit from naïve pluripotency (related to Figure 7)

A) Representative example of a bridge laser ablation experiment where the midbody remains connected to one of the daughter cells after ablation. Cells are labeled with SIR-Tubulin. Ablation is highlighted with the red thunderbolt and the location of the midbody with a pink arrowhead. One Z-plane is shown. Scale bar: 10 μm. B) Plot showing REX1-GFP mean intensity for the ablated cells pictured in (A) as a function of time. D2 is the daughter cell retaining the midbody. 0h: time of 2i/LIF removal. Lines

are fitting curves and the time of REX1-GFP downregulation is determined from the first inflexion point (see Methods).

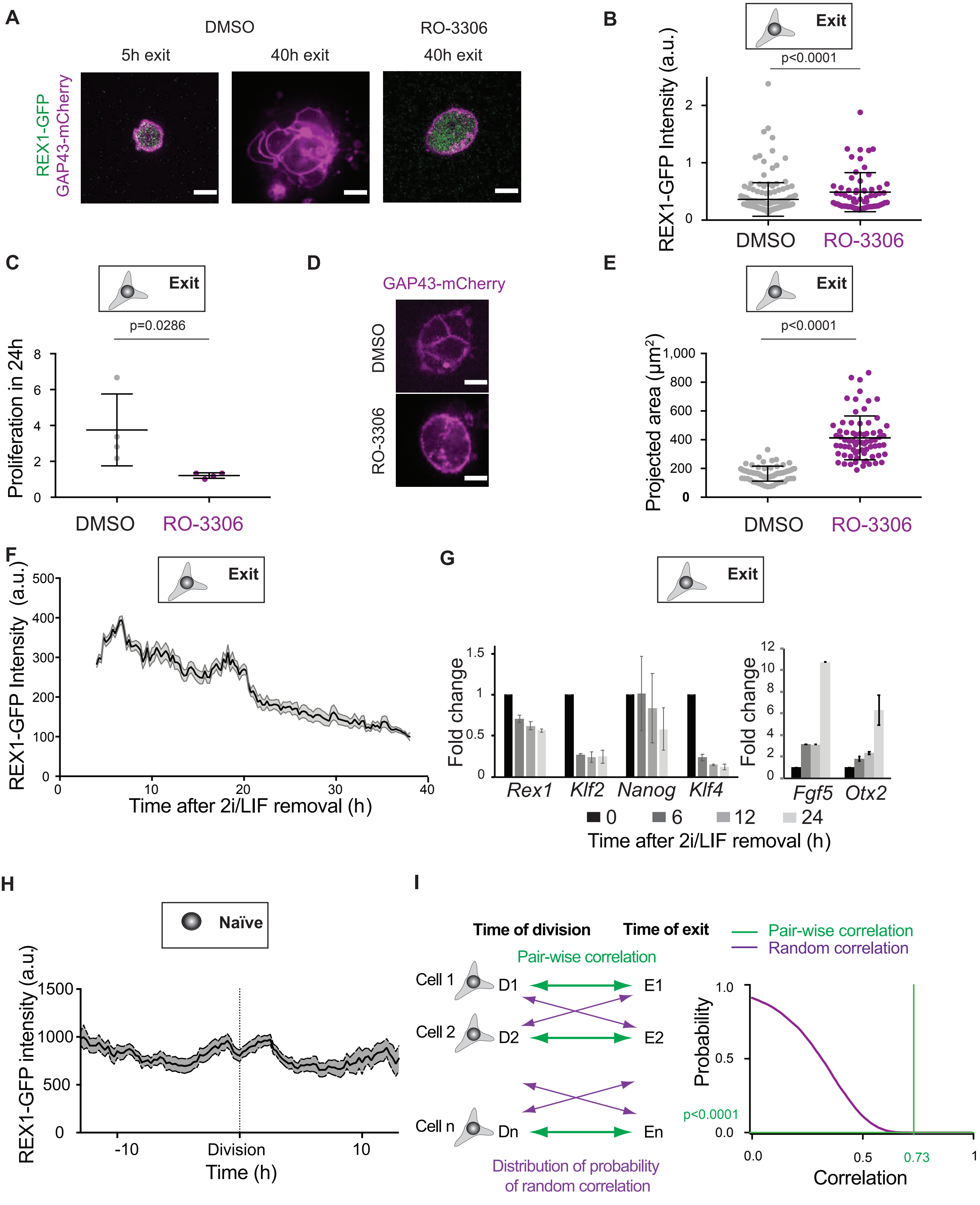

A
DMSO
RO-3306
5h exit
40h exit
40h exit
REX1-GFP
GAP43-mCherry
B
Exit
p<0.0001
REX1-GFP Intensity (a.u.)
DMSO
RO-3306
C
Exit
p=0.0286
Proliferation in 24h
DMSO
RO-3306
D
GAP43-mCherry
DMSO
RO-3306
E
Exit
p<0.0001
Projected area (μm²)
DMSO
RO-3306
F
Exit
REX1-GFP Intensity (a.u.)
Time after 2i/LIF removal (h)
G
Exit
Fold change
Rex1
Klf2
Nanog
Klf4
Fgf5
Otx2
0
6
12
24
Time after 2i/LIF removal (h)
H
Naïve
REX1-GFP intensity (a.u.)
Division
Time (h)
I
Time of division
Time of exit
Pair-wise correlation
Cell 1
Cell 2
Cell n
D1
D2
Dn
E1
E2
En
Distribution of probability of random correlation
Pair-wise correlation
Random correlation
Probability
p<0.0001
0.73
Correlation

Figure S1

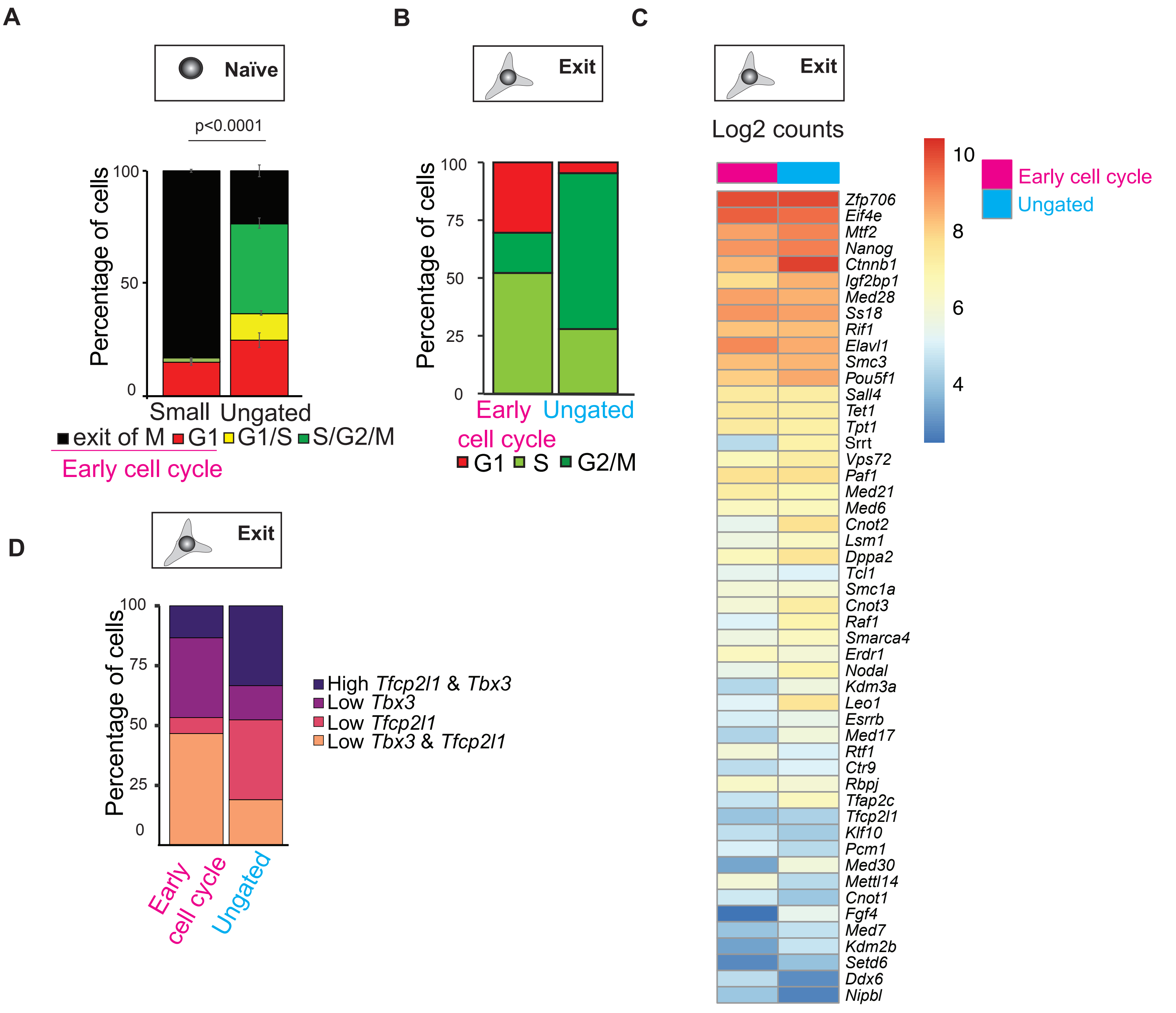
A
Naïve
p<0.0001
Percentage of cells
100
50
0
Small
Ungated
exit of M
G1
G1/S
S/G2/M
Early cell cycle
B
Exit
Percentage of cells
100
75
50
25
0
Early
cell cycle
Ungated
G1
S
G2/M
C
Exit
Log2 counts
10
8
6
4
Early cell cycle
Ungated
Zfp706
Eif4e
Mtf2
Nanog
Ctnnb1
Igf2bp1
Med28
Ss18
Rif1
Elavl1
Smc3
Pou5f1
Sall4
Tet1
Tpt1
Srrt
Vps72
Paf1
Med21
Med6
Cnot2
Lsm1
Dppa2
Tcl1
Smc1a
Cnot3
Raf1
Smarca4
Erdr1
Nodal
Kdm3a
Leo1
Esrrb
Med17
Rtf1
Ctr9
Rbpj
Tfap2c
Tfcp2l1
Klf10
Pcm1
Med30
Mettl14
Cnot1
Fgf4
Med7
Kdm2b
Setd6
Ddx6
Nipbl
D
Exit
Percentage of cells
100
75
50
25
0
High Tfcp2l1 & Tbx3
Low Tbx3
Low Tfcp2l1
Low Tbx3 & Tfcp2l1
Early
cell cycle
Ungated

Figure S2

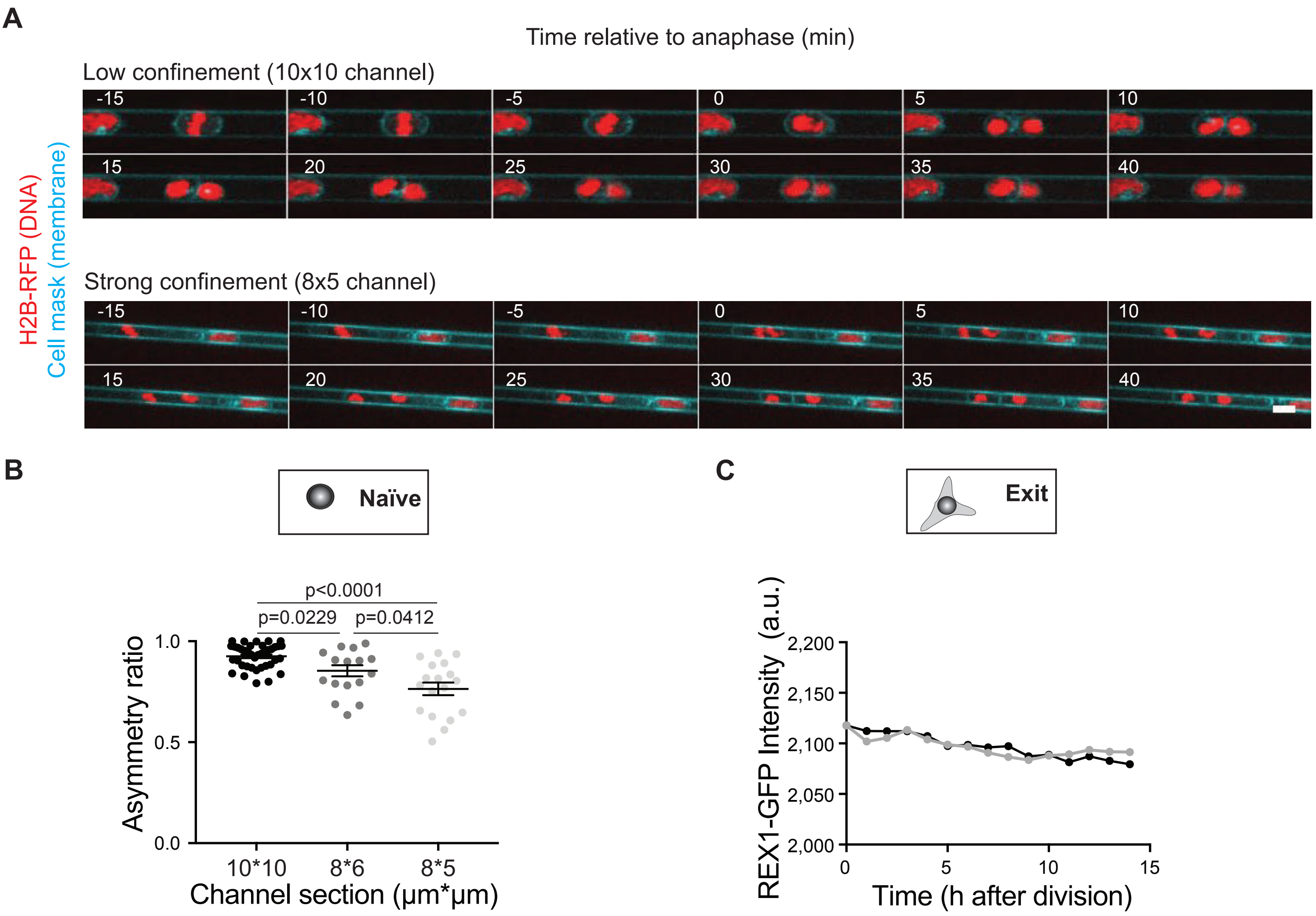

A
Time relative to anaphase (min)
Low confinement (10x10 channel)
-15
-10
-5
0
5
10
15
20
25
30
35
40
H2B-RFP (DNA)
Cell mask (membrane)
Strong confinement (8x5 channel)
B
Naïve
p<0.0001
p=0.0229
p=0.0412
Asymmetry ratio
1.0
0.5
0.0
10*10
8*6
8*5
Channel section (µm*µm)
C
Exit
REX1-GFP Intensity (a.u.)
2,200
2,150
2,100
2,050
2,000
Time (h after division)

Figure S3

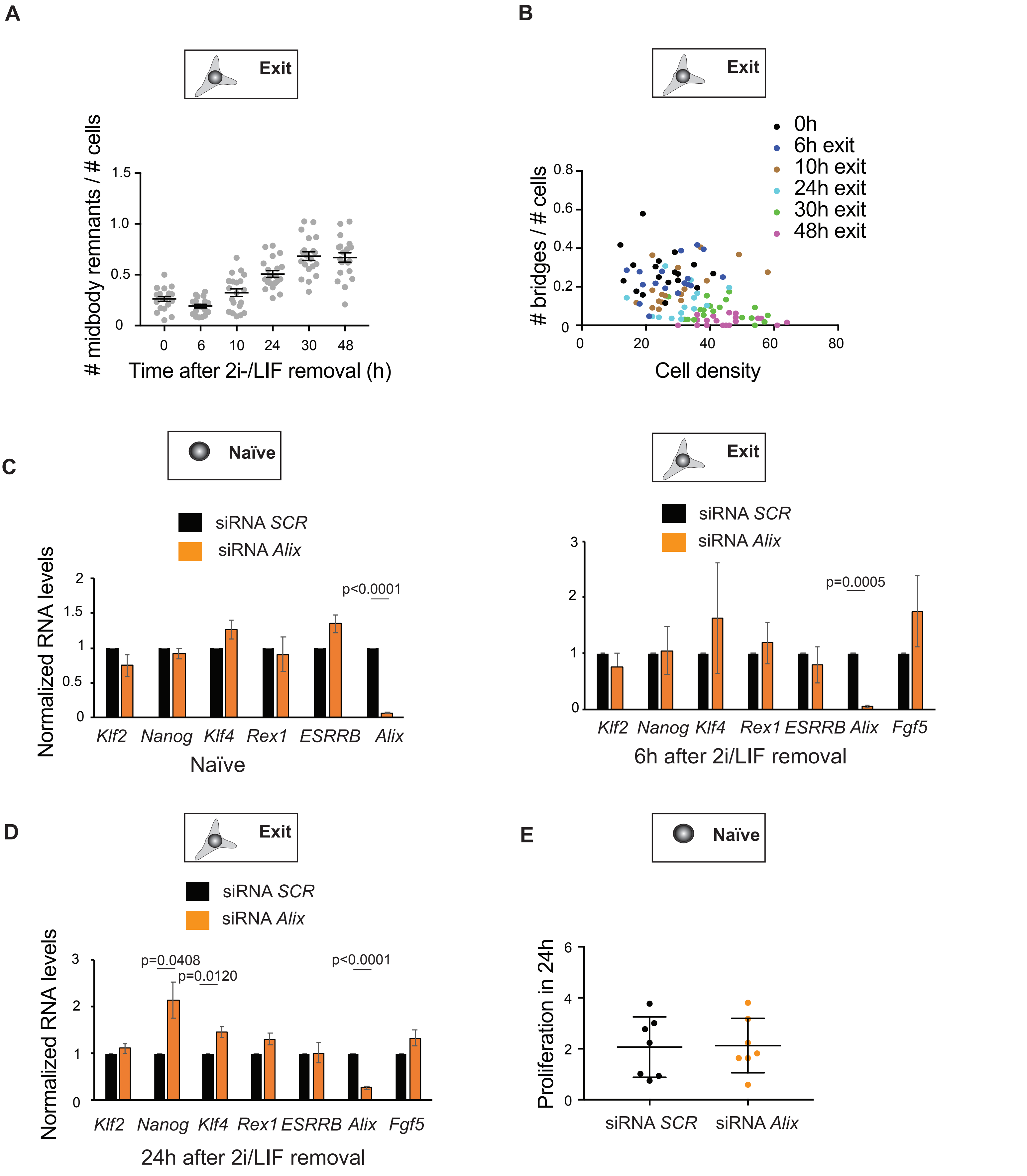
A
B
Exit
midbody remnants / # cells
Time after 2i-/LIF removal (h)
bridges / # cells
Cell density
0h
6h exit
10h exit
24h exit
30h exit
48h exit
C
Naïve
siRNA SCR
siRNA Alix
Normalized RNA levels
p<0.0001
Klf2 Nanog Klf4 Rex1 ESRRB Alix
p=0.0005
Klf2 Nanog Klf4 Rex1 ESRRB Alix Fgf5
6h after 2i/LIF removal
D
E
p=0.0408
p=0.0120
24h after 2i/LIF removal
Proliferation in 24h

Figure S4

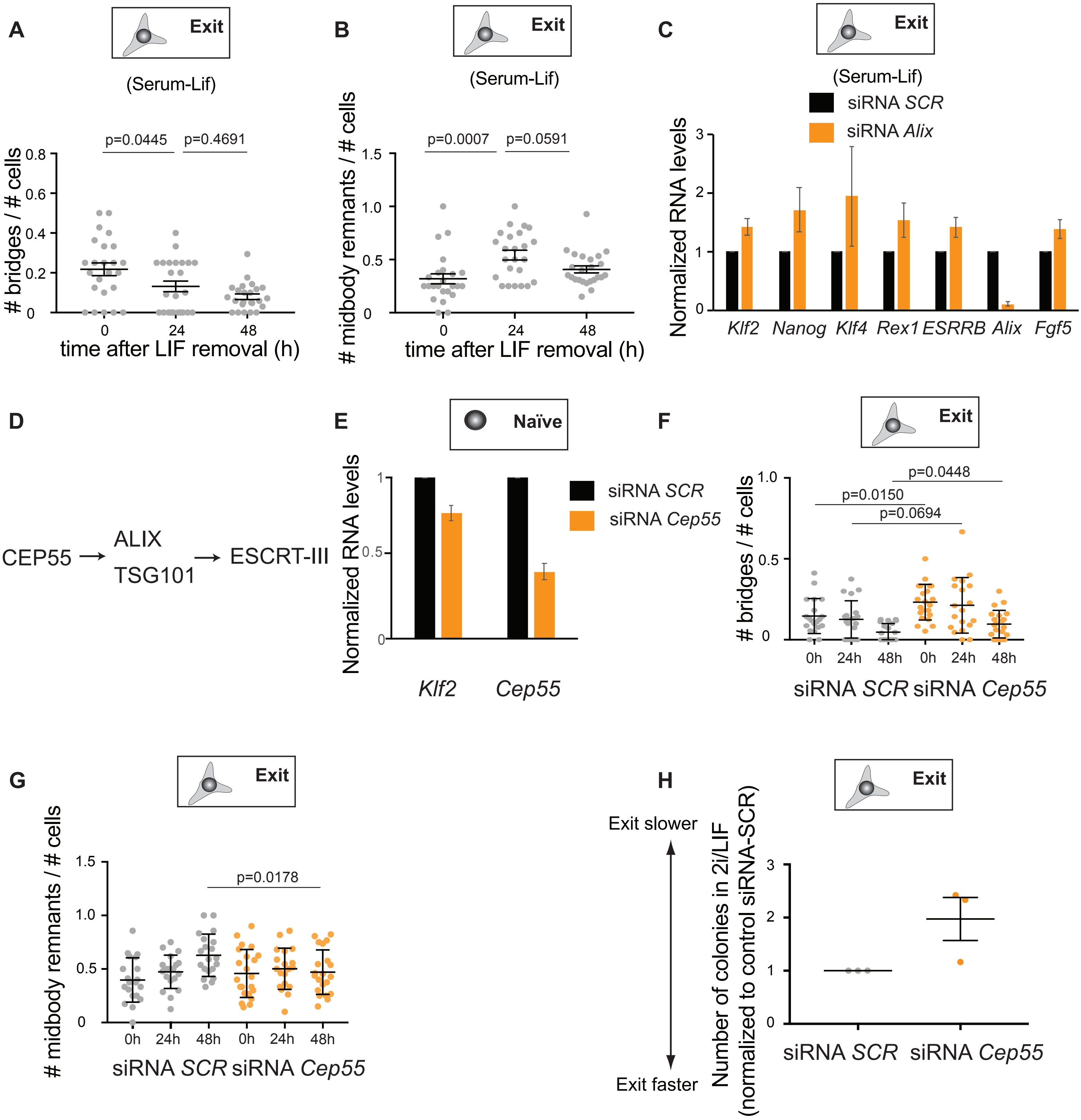
A
Exit
(Serum-Lif)
p=0.0445
p=0.4691
# bridges / # cells
time after LIF removal (h)
B
Exit
(Serum-Lif)
p=0.0007
p=0.0591
# midbody remnants / # cells
time after LIF removal (h)
C
Exit
(Serum-Lif)
siRNA SCR
siRNA Alix
Normalized RNA levels
Klf2
Nanog
Klf4
Rex1
ESRRB
Alix
Fgf5
D
CEP55 → ALIX TSG101 → ESCRT-III
E
Naïve
siRNA SCR
siRNA Cep55
Normalized RNA levels
Klf2
Cep55
F
Exit
p=0.0448
p=0.0150
p=0.0694
# bridges / # cells
0h 24h 48h 0h 24h 48h
siRNA SCR siRNA Cep55
G
Exit
p=0.0178
# midbody remnants / # cells
0h 24h 48h 0h 24h 48h
siRNA SCR siRNA Cep55
H
Exit
Exit slower
Exit faster
Number of colonies in 2i/LIF (normalized to control siRNA-SCR)
siRNA SCR
siRNA Cep55

Figure S5

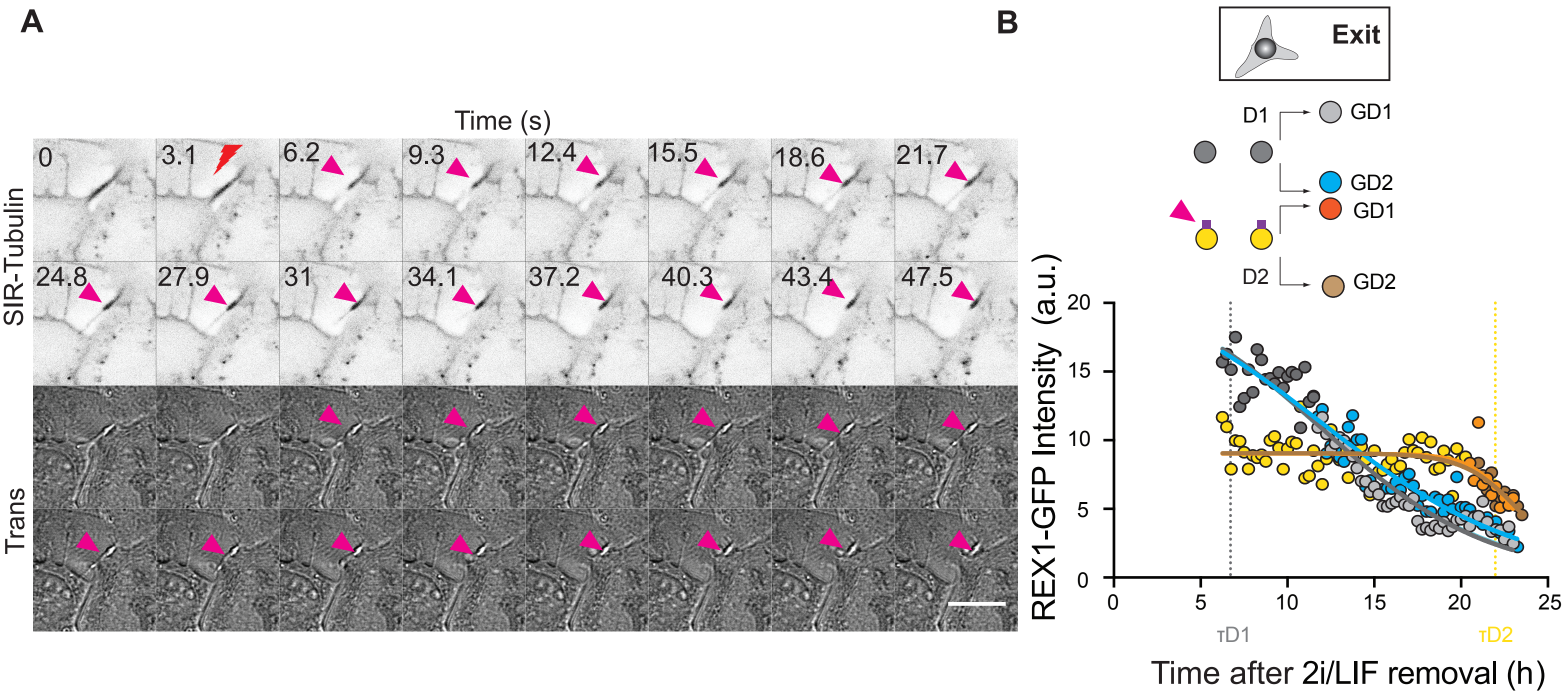
A
Time (s)
SIR-Tubulin
Trans
0
3.1
6.2
9.3
12.4
15.5
18.6
21.7
24.8
27.9
31
34.1
37.2
40.3
43.4
47.5
B
Exit
D1
GD1
GD2
GD1
D2
GD2
REX1-GFP Intensity (a.u.)
Time after 2i/LIF removal (h)
τD1
τD2

Figure S6
